# Understanding the well-being of residents in Chinese Continuing Care Retirement Communities—a case of Shanghai

**DOI:** 10.3389/fpubh.2024.1457022

**Published:** 2024-10-04

**Authors:** Xuechun Wang, Bo Xia, Martin Skitmore, Kristy Volz, Bodi Shu

**Affiliations:** ^1^School of Architecture and Built Environment, Queensland University of Technology (QUT), Brisbane, QLD, Australia; ^2^Faculty of Society and Design, Bond University, Robina, QLD, Australia

**Keywords:** Continuing Care Retirement Communities, psychological health, physical health, Ryff Scales of Psychological Well-Being (RPWB), 36-item Short Form Survey (SF-36), wellbeing, China

## Abstract

**Introduction:**

China is one of the world’s fastest-aging countries. Continuing Care Retirement Communities (CCRCs) have emerged as a viable option for accommodating and serving older adults. However, Chinese CCRCs are still in the early stages, and comprehensive research on resident well-being is still deficient. The study aims to assess how well residents in CCRCs are faring in terms of their psychological and physical health, considering China’s aging population and changing societal structures.

**Methods:**

After a thorough literature review to pinpoint relevant well-being measures in psychological and physical health, the study implemented a survey to capture residents’ experiences and perceptions, and subsequently analyzed how well-being correlates with demographic characteristics.

**Results and discussion:**

The results show that while Chinese CCRCs can enhance residents’ well-being through personalized care and social activities, challenges such as psychological distress and declining physical health remain. Demographic factors, including living situation and length of stay, also affect residents’ well-being. The study emphasizes the importance of ongoing research and evaluation to guide evidence-based practices and improve CCRCs continuously. Overall, it offers a comprehensive analysis of the wellbeing of Chinese CCRCs residents, shedding light on both psychological and physical health aspects and providing valuable insights for enhancing CCRCs design, implementation, and evaluation in China and elsewhere.

## 1 Introduction

The global population is rapidly aging, with China being one of the fastest-growing nations (1). This demographic shift presents both challenges and opportunities for public health and socioeconomic development. China must develop an integrated solution that addresses older people’s health and social needs, offering easy access to healthcare and age-friendly placement, as per recommendations from the World Health Organization (2).

China’s growing number of older adults necessitates the creation of age-friendly communities with integrated support for housing and healthcare. This is due to societal shifts from extended family systems, where younger generations traditionally cared for older parents, to now relying on commercially provided care services. A trend that has led to the ‘empty-nest older people’ phenomenon (3), as young people move to major cities for work.

Continuing Care Retirement Communities (CCRCs) are a growing sector offering integrated residential and care solutions for aging populations, primarily through market-driven mechanisms (4). CCRCs, originally from the United States, are residential or life-care communities for older adults, offering 24-h healthcare, security, social activities, dining options, housekeeping, and wellness programs (5). In contrast to their counterparts in the United States, the term “CCRCs” in China broadly and generally refers to commercial senior care facilities. These facilities are often manifested as newly developed communities offering comprehensive services that include housing, senior care, and nursing. Such communities might be designed as mixed-age environments, where a portion of units is specifically adapted for older adults, or as age homogeneity institutions that focus solely on the needs of older adults for self-care, caregiving, and assistance. Some CCRCs integrate “embedded” aged care services within the broader neighborhood to provide living and health care services (6). It is well-recognized that the market for CCRCs in China is extensive, particularly in such major cities as Guangzhou, Shanghai, and Beijing. Moreover, these facilities are predominantly aimed at affluent older individuals who have substantial disposable incomes and welfare benefits (4).

Studies indicate that CCRCs can significantly enhance residents’ well-being (7). The term well-being is defined as a multidimensional concept that encompasses an individual’s overall feelings about their life situation throughout their lifespan (8). CCRCs can improve the well-being of residents by providing personalized care plans and specialized services, such as daily living assistance, rehabilitation facilities, and health maintenance support. Such communal spaces as libraries, green spaces, and social centers encourage social connections and residents’ sense of belonging (9, 118). The well-being of older adults is influenced by environmental, social, and service-related factors (10), making CCRCs well-equipped to meet these diverse needs.

Nevertheless, the Chinese CCRCs sector is still in its early stages, lacking sufficient research and a comprehensive understanding of residents’ well-being. Challenges include psychological distress, physical health regression, and social disengagement-related inconveniences, according to Zhang and Zhang (11).

Furthermore, the lack of consensus on measuring well-being is exacerbated by the focus on single dimensions such as energy and vitality (12), ‘affect balance’ (13), self-esteem (14), depression (15), attitudes to aging (16), and satisfaction with life (17). A holistic well-being assessment requires more sophisticated multidimensional scales that include physical and psychological health, emotional well-being, and social functioning.

Therefore, this paper presents the domains of well-being and associated measures, focusing on a multidimensional approach to evaluating well-being, with the ultimate purpose of revealing the well-being of older adults in CCRCs. To achieve this, the study began with a thorough literature review on well-being measurement to identify the most appropriate one for Chinese CCRCs. Following this, a questionnaire survey was conducted to assess well-being comprehensively across six domains of psychological health and four domains of physical health. The study then analyzed the well-being status and explored the connections between these well-being measures and demographic variables, such as living status and length of stay, to better understand their potential interconnections. It provides valuable insights into the well-being of CCRCs residents, identifying areas that require further attention and guiding targeted interventions to enhance the overall well-being of different resident groups.

## 2 Methods

**Figure 1:**
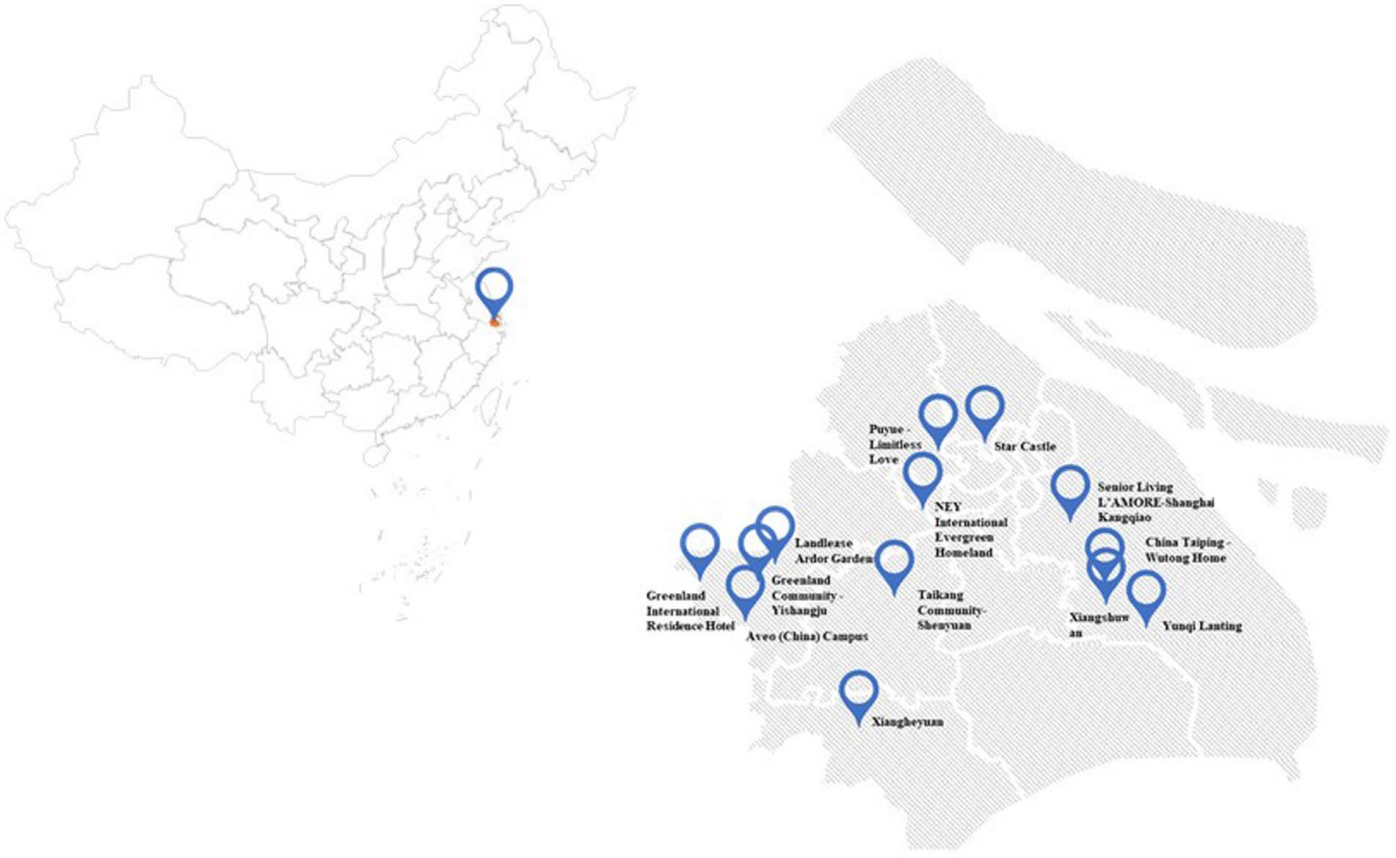
Distribution of 13 CCRC Sites in Shanghai, China.

To investigate the well-being of CCRCs residents, an in-depth survey was conducted of 13 CCRCs located in Shanghai, China (Figure 1). The entire process was methodically organized, including reviewing and selecting appropriate well-being measurement scales, developing and localizing the questionnaire, obtaining ethics approval, identifying and consulting with survey sites, distributing and collecting questionnaires, scanning and digitizing the data, and conducting a detailed data analysis.

### 2.1 Conceptualizations of well-being and dimensions of well-being scales

Initially, a thorough review of well-being scales was undertaken. The concept of “well-being” remains elusive, dating back to the ancient Greeks (18). Various terminologies, including “subjective well-being,” “adjustment,” “life satisfaction,” “happiness,” “quality of life,” “psychological health,” “wellness” and “morale” have been used to describe different aspects of well-being (19). There are ongoing discrepancies in determining the best method to measure essential aspects of well-being and optimal life functioning.

To obtain comprehensive search results, the aforementioned eight conceptual phrases were individually employed as the basis for searches in both Chinese and English databases, including Web of Science, John Wiley, JSTOR, and CNKI. Understanding the strengths and limitations of various measurements, such as unidimensional, bidimensional, or multidimensional, is crucial, as the choice of measurement depends on the research question being studied (20). Therefore, for each study, the sources and their measurement domains were reviewed separately to identify scales that could comprehensively explore the overall well-being of older adults. This has been addressed in our previous research (6), laying the foundation for the subsequent evaluation of the structure of well-being.

Evaluating the structure is equally crucial in selecting the appropriate scales. The structure of well-being, derived from various approaches, such as “hedonic,” “eudaimonia,” “Quality of Life (QoL),” and “Wellness,” is crucial in selecting appropriate scales.

*Hedonic* approaches focus on pleasure and happiness, with subjective well-being being the predominant model (21). It includes life satisfaction, the absence of negative emotions, and the presence of pleasant emotions (17). Advocates of this viewpoint typically define well-being by considering all three of these constructs. In contrast, *Eudaimonic* approaches to well-being suggest that psychological health is achieved through fulfilling one’s capabilities, functioning optimally, or actualizing one’s inherent essence (21). They focus on a broader range of life domains, such as the psychological well-being model (22), which consists of six components: positive relationships with others, autonomy, environmental mastery, purpose in life, self-acceptance, and personal growth.

Additionally, the *Quality of Life (QoL)* model (21) is a widely used approach to understanding well-being, encompassing physical, psychological, and social functioning. It is often used interchangeably with hedonic or eudaimonic models (23), and is influenced by medicine, sociology, and psychology. The QoL model is commonly used in medical studies and is shaped by such diverse fields as medicine, sociology, and psychology (24). Furthermore, *Wellness* is a fourth approach to understanding well-being (21), with some researchers using the terms interchangeably. Wellness, a concept similar to eudaimonic approaches, emphasizes optimal functioning and a comprehensive approach to maximizing an individual’s potential, encompassing all aspects of health and functioning, including physical, spiritual, and integrated personality (25). Wellness approaches vary in component, but they all emphasize that being well is more than just the absence of illness. Experts believe wellness should be a holistic lifestyle (25).

Given the multifaceted nature of well-being, multidimensional well-being scales, which evaluate various aspects of well-being by individual domains, offer a more comprehensive understanding of well-being than unidimensional scales (26). Of these scales, the most frequently encountered domains were Affect, Social relations, Physical health, Life satisfaction, Meaning/Achievement, and Spirituality (6). Hence, the assortment scales outlined below are capable of covering the previously identified domains: the Social Production Function Instrument for the Level of Well-being (SPF-IL) (27), Ryff’s Scales of Psychological Well-being (RPWB) (119), the Multi-dimensional Personality Questionnaire Well-being Scale (MPQ-WB) (28), the Multicultural Quality of Life Index (MQLI) (29), and the Life Satisfaction Rating Scale (LSR) (30).

Assessing the well-being of older adults is crucial due to their unique needs and experiences. The WHO defines “well-being” as physical, psychological, and social functioning (6), and the RPWB-54 and SF-36 scales can be used to evaluate the overall well-being of older individuals. The Ryff Scales of Psychological Well-Being (RPWB) is an effective model that aligns with World Health Organization (WHO) criteria, except for physical health. An additional measure, the 36-item Short Form Survey (SF-36), is added to the RPWB to understand and evaluate well-being fully. The SF-36 is particularly effective in assessing physical health in older adults, and its widespread use in research has proven its value in accurately evaluating these outcomes (31). The combined application of RPWB-54 and SF-36 scales for measuring well-being is deemed appropriate for this study.

The RPWB-54 scale was chosen for the present study due to its unique advantages, covering six domains related to psychological well-being: Self-acceptance (SA), Positive Relations with Others (PR), Autonomy (AU), Environmental Mastery (EM), Purpose in life (PL), and Personal Growth (PG) (32). By integrating subjective experiences and evaluations of well-being, RPWB offers a broader understanding of older adults’ psychological health, moving beyond traditional pathology-based definitions and aligning with the research objectives (33). Previous studies have used various versions of the RPWB scales, such as the 18-item version, but poor model fit, and consistency led to their exclusion (34). Pedhazur and Schmelkin (35) suggested excluding further items in the 120-item version for a shorter, psychometrically robust measure. Van Dierendonck and Lam (36) proposed shorter scales, such as 42-item, 54-item, and 84-item versions, as viable alternatives. Considering these factors, the 54-item RPWB Ryff scale (RPWB-54) was selected for this study based on the physical capabilities of older adult participants, considering their physical capabilities.

The SF-36 is a comprehensive health assessment tool that effectively captures health-related well-being, covering both physical and psychological health domains (37). It assesses eight domains: physical functioning (PF), role-physical (RP), bodily pain (BP), general health perceptions (GH), vitality (VT), social functioning (SF), role-emotional (RE), and mental health (MH). Its components assess two principal dimensions: the Physical Component Summary (PCS) and the psychological aspect represented by the Mental Component Summary (MCS) (37). The SF-36’s flexibility allows for the independent use of physical and psychological components within the originator’s guidelines, making it an adaptable supplement to scales such as the RPWB-54.

### 2.2 Questionnaire development

Afterward, a questionnaire was designed to assess overall well-being. The questionnaire was divided into two primary sections. The initial section gathered information on the respondents’ demographics (including age, gender, marital status, income over the past year, educational attainment, household registration type, community location, present living conditions, and duration of residence); the second section evaluated well-being, using the RPWB-54 to gauge psychological health and the SF-36 to measure physical health, offering a comprehensive overview of the residents’ overall well-being. The full English questionnaire is available in Appendix A. With the support of, Carol D. Ryff and her research team, developers of the RPWB-54 scale, supplied a Chinese version. This version underwent localization to improve clarity for older Chinese respondents, and the authors unanimously approved the revised phrasing of this study.

### 2.3 Site selection and questionnaire distribution strategy

Subsequently, sites for questionnaire distribution were identified. Due to three pivotal considerations, Shanghai was chosen as the site for data collection of CCRCs. Primarily, the city’s substantial aging demographic, comprising 5.82 million individuals aged 60 years and above (23.4% of the total population), establishes Shanghai as a quintessential city for the examination of CCRCs and the well-being of older adults. Secondly, Shanghai’s policy environment has significantly encouraged investment in the development of CCRCs, particularly following the 2005 “9,073 pattern” policy, which designates care for 90% of older adults at home, 7% through community-integrated care support, and 3% in institutional care facilities. Shanghai’s first CCRC project, Kang-Qiao Community, was established in 2008. Lam and Yan (38) summarized Shanghai’s eleven CCRCs in their previous study, and our research has supplemented this by identifying seven more, as detailed in Appendix B, such as Landlease Ardor Gardens, NEY International Evergreen Homeland. This diversity supplied a rich range of data sources, facilitating comprehensive data collection. Lastly, Shanghai’s pioneering role in the CCRCs sector development, evidenced by its robust economic status and significant capital infusion from commercial developers, affords invaluable insights into potential market expansion growth.

Following ethical approval and detailed consultations with CCRCs community leaders, collaborative arrangements were established with 13 representative CCRCs in Shanghai for questionnaire response collection, focusing on independent older adults. During the ethics approval process, a comprehensive set of materials was submitted, including the study content, consent forms, questionnaire samples, recruitment advertisements, agreements for volunteers, expert interview materials, and other related documents. These materials received approval from the University Human Research Ethics Committee (UHREC) on October 22, 2021, ensuring the study’s adherence to ethical standards. Subsequently, leaders from CCRCs in Shanghai were initially contacted via email, providing comprehensive materials such as proof of ethical approval and questionnaire samples. The emails detailed the study’s objectives, scope, and significance, emphasizing the importance of protecting participant rights, assuring participant anonymity, and highlighting the community’s role in contributing to the study. Although specific community-level sampling criteria were not applied, a more extensive and varied sample was still included in the selection of CCRCs to better capture the overall characteristics of Shanghai’s CCRCs landscape. Following preliminary agreements with some CCRCs, well-trained volunteers, including community managers or higher-ranking officials, visited Shanghai to meet community leaders in person. These discussions covered reward mechanisms for questionnaire participants and the questionnaire distribution strategy. Based on community managers’ suggestions, the questionnaire wording was localized for better comprehension by older adults, and the layout and font size were adjusted for readability. Safety measures were also discussed to minimize direct contact during questionnaire distribution and collection, ensuring the health and safety of older adults amid the pandemic. Additionally, detailed training was provided to community staff to ensure familiarity with the questionnaire content and distribution procedures. This training also included guidance on assisting older adults in completing the questionnaire, ensuring the accuracy and completeness of the responses. This extensive coverage enhances the study’s representativeness and offers a comprehensive understanding of urban CCRCs’ overall conditions.

These 13 CCRCs fall into three distinct categories: all-age institutions, age-homogeneity institutions, and embedded older care facilities within their neighborhoods. Within each category, the CCRCs exhibit notable diversity in their investment, development, operational models, and service offerings. Appendix C provides an overview of the diversity, characteristics, and management of the 13 selected CCRCs.

#### 2.3.1 Age-homogeneity institutions

In the category of age-homogeneity institutions, several CCRCs projects are invested in by Fortune Global 500 companies, such as Taikang Group, Lendlease Group, and Greenland Group. Taikang Community has pioneered the integration of pension insurance, health insurance, and asset management with CCRCs communities and medical systems, utilizing a membership card model to grant residency rights. It is China’s first insurance-based senior care community approved by the China Insurance Regulatory Commission and the largest in Shanghai and East China. Notably, the “membership card” is emerging as a unique model currently prevalent in Chinese CCRCs and widely adopted by major communities. This model allows residents to obtain living and service rights within the community by purchasing high-value membership cards, which are typically inheritable and transferable. Depending on the community’s regulations, these cards may offer varying levels of care and services. This model aids in rapid capital recovery during the initial stages and ensures sustained, stable cash flow, thus becoming a major profit model for many Chinese CCRCs.

Lendlease Ardor Gardens, entirely developed by Lendlease Group, represents its first integrated development model in China, offering hotel-style and membership-based care services for older people. The Greenland International Residence Hotel leverages a membership card to the Greenland Community, Yishangju, to offer “migratory bird” care services nationwide. Additionally, several projects feature international collaborations. NEY International Evergreen Homeland, in partnership with prominent global medical and care institutions, is situated on Shanghai’s first paid-transfer land designated for retirement communities and is recognized as the city’s first green, sustainable CCRC. Star Castle, a joint venture between Fosun Group and Fortress Investment Group, is the first CCRC in Shanghai with foreign investment and older adult care licenses, introducing an all-inclusive rental model and leveraging Fortress’s extensive operational experience from the United States. Puyue—Limitless Love is an open CCRCs community that integrates urban areas with holistic care, combining the CCRCs model with a “Zen” culture hotel for younger generations to promote intergenerational living. Fully funded and operated CCRCs include Xiangshuwan, developed by a subsidiary of Shanghai Wanfeng Group, utilizing a membership card model, and Xiangheyuan, entirely funded by Shanghai Fuyi Elderly Care Co., Ltd., operating on a leasing model with fees based on room type and rental period.

#### 2.3.2 All-age institutions

Within the all-age institution category, AVEO CAMPUS, formed through a joint venture between FKP Property Group and China Tiandi Holdings, leverages a combined sales and leasing profit model. This project integrates over 30 years of AVEO’s experience in high-end senior living operations in Australia, primarily providing comprehensive services for older residents while also offering amenities such as hotels and kindergartens for visitors and residents’ families. China Taiping—Wutong Home, backed by Fortune Global 500 company China Taiping Insurance Group, employs a diversified profit strategy through the sale of insurance products, membership cards, and older adult care services. It uniquely implements a “migratory bird” model, allowing residents to live in different community locations across 16 cities depending on the season. Greenland Community—Yishangju employs a membership card model to mitigate initial funding pressures. It integrates health tourism into its service offerings, and in its third phase, development plans include long-term rental apartments and kindergartens to establish a comprehensive all-age community. Yunqi Lanting, independently developed by Dongju Enterprise, combines membership cards and leasing in its profit model, offering flexible short-term rental services and family rooms to accommodate diverse resident needs.

#### 2.3.3 Neighborhood-embedded living and care institutions

In the category of embedded older care facilities, Senior Living L’AMORE-Shanghai Kangqiao serves as a prominent example. Funded by Sino-Ocean Group and operated in collaboration with Emeritus and Meridian Senior Living (MSL), this project adopts a light asset model by leasing and converting buildings into senior apartments with integrated care services. The building areas, project capacities, and resident populations of these CCRCs vary significantly. Some CCRCs encompass large areas and can accommodate 1,000 of residents, while others are smaller in scale, serving specific demographic groups. This diversity enables each community to tailor its services and facilities to meet the unique needs of its residents. By examining a diverse set of CCRCs, the study enhances the generalizability of its results, ensuring they are applicable to a wider range of CCRC types and demographic groups.

### 2.4 Questionnaire distribution, data collection, and analysis

Following this, questionnaires were distributed and collected at the selected sites. The survey primarily targeted older adults who live independently. Given the significant resources it would require and the varying willingness to participate, a population census sampling approach was not adopted. Instead, voluntary participation was encouraged for all eligible individuals. As residents with varying levels of independence are housed in different buildings or floors for easier management, the distribution and collection of questionnaires were conducted in a fair, just, and transparent manner to ensure equal participation opportunities for all eligible residents. The data was collected through the distribution of paper questionnaires, augmented by online links and Quick Response (QR) codes through the Questionnaire Star platform-a tool of repute for academic research within China. This process, supported by the collaboration of community managers, used varied distribution strategies across different communities to ensure widespread participation. For instance, in 12 CCRCs, announcements were made inviting residents to collect questionnaires from communal dining areas or senior centers. Alternatively, community managers and volunteers facilitated sessions wherein residents congregated to complete the questionnaires at designated times and locations. Notably, at the China Taiping-Wutong Home, a singular CCRCs adopting a distinct approach, caregivers facilitated older residents’ access to online questionnaires via the Questionnaire Star platform, where the residents either independently completed the questionnaires or were assisted by caregivers who verbalized the questions, ensuring all residents could adapt to and were not hindered by this technological method. Because of the impact of COVID-19, volunteers had to avoid direct interaction with older adults and only offered indirect support in these CCRCs. Each questionnaire was accompanied by an introductory letter outlining the research objectives and a certificate confirming ethics approval.

To incentivize participation and ensure the collection of high-quality responses, residents were provided with modest rewards. A baseline reward of 20 RMB was given for completing the questionnaire, while those who also submitted detailed retirement plans, identified potential conflicts, and proposed solutions were awarded 30 RMB. This incentive scheme was consistently applied across all CCRCs, except for Xiangheyuan Home Care Community, which offered eight eggs and 10 RMB to align with resident preferences. The majority of participants spent more than 3 h on the questionnaire, with some taking an entire morning or day to discuss and analyze the questions with their neighbors. Consultations with volunteers and community staff were limited to question interpretation, thereby maintaining the independence and accuracy of their responses.

One thousand, one hundred and seven (1,107) questionnaires were successfully collected between November 2021 and May 2022. Table 1 summarizes the distribution and proportion of questionnaire sources. It is important to clarify that the volume of questionnaire responses collected does not necessarily correlate with the resident count of the communities. This variation is primarily due to differences in residents’ willingness to participate and the safety challenges encountered during data collection. For instance, despite our implementation of incentive measures and the provision of comprehensive documentation, residents’ levels of trust and interest in participating in the survey varied. Additionally, at Landlease Ardor Gardens, questionnaire distribution was momentarily suspended due to sudden health and safety precautions during the COVID-19 pandemic.

**Table 1:** Distribution and proportion of questionnaire sources.

CCRCs	Number of questionnaires	Proportion
Landlease Ardor Gardens	2	0.2%
NEY International Evergreen Homeland	11	1.08%
Senior Living L’AMORE-Shanghai Kangqiao	30	2.95%
Star Castle	40	3.93%
Greenland Community—Yishangju	50	4.92%
Greenland International Residence Hotel	53	5.21%
Yunqi Lanting	80	7.87%
Xiangshuwan	86	8.46%
Xiangheyuan	100	9.83%
Puyue—Limitless Love	102	10.03%
China Taiping—Wutong Home	138	13.57%
Aveo (China) Campus	157	15.44%
Taikang Community-Shenyuan	168	16.52%

The quantity of questionnaire responses is independent of the community’s resident population. The survey was conducted with the assistance of community managers. The questionnaire was widely distributed among older adults in certain communities, such as at Taikang Community-Shenyuan. However, in some communities, the questionnaire distribution was temporarily suspended due to various reasons, including considerations for the health and safety of the older adult population, as observed at Landlease Ardor Gardens.

Post-collection, volunteers scanned and transmitted the questionnaires to the Australian research team, where the data was digitized via Questionnaire Star to facilitate unified analysis with China Taiping-Wutong Home’s platform. To ensure data entry accuracy, a double-entry process was implemented: Researcher A initially input the data into Questionnaire Star, after which Researcher B verified the entries against the scanned originals. This role reversal was repeated for thorough cross-checking. Additionally, to ensure data integrity and prevent loss, multiple backup measures were employed, with digitized data stored in Unique RDSS Space, local hard drives, and Questionnaire Star, while the original paper questionnaires were retained. Data preprocessing adhered to Little and Rubin’s (39) guidelines, excluding responses with over 10% missing data and considering the remaining responses as valid. This preprocessing was conducted using Excel.

Finally, the collected data underwent thorough analysis using IBM SPSS Statistics for Windows (version 27.0). Descriptive statistical methods were used to summarize the nine primary demographic attributes of the respondents, including age, gender, marital status, total income over the past 12 months, education level, Hukou type (Hukou is a household registration system used in Mainland China), community location, living status, and length of stay, to establish a comprehensive understanding of the demographic profile of CCRCs residents. By analyzing 10 domains—four physical and six psychological health domains—this approach intended to offer an extensive overview of residents’ health conditions and a detailed examination of each domain’s specifics.

Differential analysis was then applied to investigate the relationship between residents’ levels of well-being and demographic characteristics. To facilitate this analysis, well-being scores were categorized into distinct groups. The lowest quartile (less than 25) was excluded due to an absence of data within this range. The remaining categories were reclassified as “low well-being,” “medium well-being,” and “high well-being,” with scores below 25 indicating low well-being, 50 indicating medium well-being, and 75 indicating high well-being. Independent samples t-tests and one-way ANOVA were used to analyze potential disparities between demographic characteristics and residents’ well-being, providing deeper insights into the primary reasons behind observed variances.

## 3 Result

### 3.1 Well-being scales

We identified and summarized 44 measurement instruments of well-being, consolidating scales with the same domains but different numbers of items into a single instrument, as summarized in Table 2.

**Table 2:** Forty four measurement scales of well-being.

Author(s), year	Name of measurement scale (abbreviation)	Domains
Neugarten et al. (30)	Life Satisfaction Rating Scale (LSR)	Zest, resolution and fortitude, congruence between desired and achieved goals, positive self-concept, and mood tone
Neugarten et al. (30)	Life Satisfaction Index	Zest, congruence between desired and achieved goals, positive self-concept, affects
Bradburn (13)	Affect Balance Scale (ABS)	Positive, negative affects
Lawton (83)	Philadelphia Geriatric Center Morale Scale (PGC)	Agitation, loneliness, attitudes towards ageing
Dupuy (84)	General Well-being Schedule (GWB)	SWB, self-control, vitality, anxiety, depression, general health
Kozma and Stones (120)	Memorial University of Newfoundland Scale of Happiness (MUNSH)	Positive affect (PA), negative affect (NA), positive experience (PE), negative experience (NE)
Eysenck (121); Rosenberg (85)	OHI+ Eysenck Personality Questionnaire (EPQ) + Rosenberg’s Self-esteem Scale (RSS) + Satisfaction with Life scale (SLS)	Satisfaction with life, personal efficacy, sociability/empathy, a positive outlook, physical well-being, cheerfulness and self-esteem
Ferrans and Powers (86)	Quality of Life Index (QLI)	Family relations, health, resources, spiritual WB
Diener et al. (17)	Satisfaction with Life Scale (SWLS)	Positive affect, absence of negative affect, life satisfaction
Csikszentmihalyi and Larson (87)	Experience Sampling Method (ESM)	Affect, arousal, cognitive efficiency, motivation
Antonovsky (88)	Sense of Coherence Scale (81)	Comprehensibility, manageability, meaningfulness
Watson et al. (89)	Positive and Negative Affect Schedule (PANAS)	Positive, negative affects
EuroQOl Group (90)	EuroQOL-EQ-5D	Mobility, self-care, usual activities, pain, anxiety/depression
Tibblin et al. (91)	Gothenburg Quality of Life Measurement Scale (GQL)	Affects, social SWB, physical health, life satisfaction
Ware and Sherbourne (92)	MOS 36-Item Short Form Health Survey	Physical function, role physical, general health, social functioning, pain, mental health, vitality
Bech et al. (93)	WHO (Five) Well-being Index	Cheerful, calmness, activity, rest, interest
Ruta et al. (94)	Patient Generated Index General Well-being Scale (PGI)	Health, affects, relationships
Scheier et al. (95)	Life Orientation Test of Optimism and Pessism-Revised (LOT-R)	Optimism, pessimism
Mezzich et al. (29)	Multicultural Quality of Life Index (MQLI)	Physical SWB, psychological SWB, self-care, independent functioning, occupational functioning, interpersonal functioning, social–emotional support, community and services support, personal fulfillment, spiritual fulfillment, overall quality of life
Ryff and Keyes (32)	Ryffs Scales of Psychological Well-being (RPWB)	Self-acceptance, positive relations, autonomy, environmental mastery, purpose in life, personal growth
Watson and Clark (96)	Mood and Anxiety Symptom Questionnaire (MASQ)	General distress, anxiety, depression
Patrick et al. (28)	Multidimensional Personality Questionnaire Well-being Scale (MPQ-WB)	Social potency, achievement, social closeness, stress reaction, alienation, aggression, control, harm avoidance, traditionalism, absorption
Cummins (97)	Comprehensive Quality of Life Scale (COMQOL-A4)	Material SWB, health, productivity, intimacy, safety, place in community, affects
Kaplan et al. (98)	Quality of Well-being Scales (QWB-SA)	Health, mental health, mobility, physical activity, relationships
Whoqol Group (99)	WHOQOL	Physical health, psychological health, social relationships, level of independence, environment, spirituality
Prince et al. (100)	Euro-D	Affective suffering, motivation
Goldberg and Williams (101)	General Health Questionnaire-12 (GHQ-12)	Positive, negative affects
Hyde et al. (102)	Quality of Life Scale (CASP)	Control, autonomy, affects, self-realization
Ventegodt et al. (103)	Self-evaluation of Quality of Life (SEQOL)	Well-being, life satisfaction, happiness, family, fulfilment of needs, satisfaction with relationships, releasing life potential, objective factors, quality of life
Kahneman et al. (104)	Day Reconstruction Method (DRM)	Life satisfaction, feelings
Ingersoll-Dayton and Krause (105)	Thai Well Being Scale	Life satisfaction, relationships
Peterson et al. (106)	Orientation to Happiness Scale (OTH)	Meaning, pleasure, engagement
Nieboer et al. (27)	Social production function (SPF-IL)	Universal goals affection, behavioural confirmation, status, comfort and stimulation
Al Naser et al. (107)	Kuwaiti Raha Scale (KRS) 2010	Religiosity, happiness, stability, confidence, likable
Tennant et al. (108)	Warwick-Edinburgh Mental-Well-being Scale (WEMWBS)	Optimism, purpose in life, relaxed, cognition, relationships, feeling loves
Katerndahl and Oyiriaru (109)	Bio-psycho-socio-spiritual Inventory (BioPSSI)	Health, emotions, relationships, spirituality, life satisfaction
Rothmann and Ekkerd (110)	Perceived Wellness Survey (PWS)	Health spirituality, affects, relationships, emotions, cognition
Kahneman ans Deaton (111)	Gallup-Healthways Well-being Index (GHWBI)	Life satisfaction, emotional health, physical health, healthy behaviours, work environment, access to health care
Bringsén et al. (112)	Salutogenetic Health Indicator Scale (SHIS)	Affects, relationships
Vaingankar et al. (113)	Positive Mental Health Measurement Scale (PMH)	Coping, emotional support, spirituality, relationships, personal growth, affects
Kinderman et al. (114)	BBC Well-being Scale (BSC)	Life satisfaction, personal growth, relationships
Bann et al. (115)	Public Health Surveillance Well-being Scale (PHS-WB)	Life satisfaction, meaning in life, autonomy, competence, relatedness, affects, relationships, health, energy
Supranowicz and Paź (116)	PMSW	Physical domain, mental domain, social domain

### 3.2 Profile information of respondents

From the 1,107 senior residents across 13 CCRCs who participated in the questionnaire survey, 815 valid responses were retained after excluding those with over 10% missing data. Lee et al. (40) define demographics as population characteristics such as gender, age, and marital status. Table 3 details the demographics of the survey participants.

**Table 3:** Demographics of 815 questionnaire survey respondents.

Personal information		*N*	(%)
Age	<56	34	4.2
	56–65	67	8.3
	66–75	200	24.6
	76–85	360	44.3
	>85	151	18.6
Gender	Male	344	42.2
	Female	441	54.1
Marital status	Unmarried	15	1.9
	Married	580	72.4
	Divorce	45	5.6
	Widowed	161	20.1
Total income for the past 12 months	<60,000	131	16.1
	60,001-120,000	331	40.6
	120,001-180,000	224	27.5
	180,001-240,000	95	11.7
	>240,000	26	3.2
Education level	Junior high school and below	104	12.8
	High school or technical secondary school	248	30.4
	University	404	49.6
	Postgraduate and above	57	7.0
Hukou type	Urban Hukou	788	96.7
	Agricultural registered permanent residence	22	2.7
Community location	City	210	25.8
	Suburbs	503	61.7
	Suburban and city junction	98	12.0
Living status	Living alone	233	28.6
	With partner	548	67.2
	With others	28	3.4
Length of stay	<1 year	117	14.4
	1–3 years	271	33.3
	3–5 years	275	33.7
	5–10 years	73	9.0
	>10 years	74	9.1

The total number of respondents in each category is not 815 due to missing data for some personal information.

Most respondents (44.3%) are aged 76–85, with a gender distribution of 54.1% female and 42.2% male. The majority (72.4%) are married, with 20% being widowed. Financially, 42.4% report an annual income exceeding CNY 120,000, significantly higher than Shanghai’s average *per capita* income of CNY 78,027 in 2021 (41). Nearly half of the respondents have a university degree, followed by high school or technical secondary education (30.4%), and a minority (12.8%) with junior high school education or less.

The majority of respondents in Shanghai, comprising 96.7%, own an urban Hukou, a household registration system in China, compared to only 2.7% holding an agricultural Hukou. This demographic is crucial for understanding the experiences and perspectives of CCRCs residents.

The survey data shows that respondents are distributed across three geographical areas: 25.8% urban, 61.7% suburban, and 12% intersection of suburban and urban areas. This reflects the diverse preferences of older individuals, ranging from nature enthusiasts to urbanized socialites. Living arrangements within CCRCs are 28.6% independent, 67.2% co-reside with spouses and 3.4% share living spaces. The data shows that over half of the respondents in CCRCs have lived there for three or more years, with those over a decade comprising 9.1% of the total participants.

### 3.3 Psychological and physical well-being of CCRCs residents

Table 4 presents Ryff’s six domains of psychological well-being. The highest score was in Environmental Mastery (EM) at 74.33/100, indicating that residents feel competent in managing their environment and daily life. Self-Acceptance (SA) also scored high at 73.15/100, reflecting a positive self-view and acceptance of the past. Positive Relations (PR) scored 71.78/100, suggesting strong social connections among residents. The Purpose in Life (PL) domain scored 70.07/100, indicating an acceptable level of purpose among CCRCs residents, ranking fourth out of six. The average score of 66.40/100 in the Personal Growth (PG) domain suggests that CCRCs should focus on enhancing residents’ personal growth and addressing practical challenges. Although Autonomy (AU) scored the lowest among the psychological well-being domains, it still falls within the high scorer zone (≥50), indicating that residents maintain an acceptable level of self-determination and personal standards.

**Table 4:** Psychological health domains ranking.

Rank	Domains	Average score (out of 100)
1	Environmental mastery (EM)	74.33
2	Self-acceptance (SA)	73.15
3	Positive relations with others (PR)	71.78
4	Purpose in life (PL)	70.07
5	Personal growth (PG)	66.40
6	Autonomy (AU)	65.18
	Average score	70.15

EM refers to an individual’s ability to create settings based on their psychological state (22), reflecting the supportive environment provided by CCRCs that enables residents to maintain control over their lives (e.g., offering various activities and choices in daily routines). SA emphasizes acceptance of oneself and past life (22). The older CCRCs adults demonstrated good self-acceptance, indicating their ability to create environments where residents feel acknowledged and find peace, such as celebrating personal milestones or participating in memory sharing sessions. This encourages a sense of belonging, maintains positive self-esteem, and promotes emotional health. Additionally, the PR domain, which includes strong empathy (22), affection, and deeper interpersonal connections, was rated 71.78/100 by CCRCs residents. This score suggests that the CCRCs environment, with its communal living arrangements and shared activities, likely maintains or enhances relational qualities by providing numerous opportunities for social engagement, fostering community and enabling residents to connect with neighbors, caregivers, and visitors on an optimistic level. Examples include communal dining areas, social events, and group activities.

The PL domain, which involves having goals, intentions, and direction for a meaningful life (22), received an average score of 70.07/100 from CCRCs residents, ranking fourth out of six. As residents transition through care stages, their perceptions may change due to health status, social networks, and independence. The PL domain’s lower ranking suggests a need for CCRCs to implement measures to help residents find new passions or continue pursuing long-held interests, reinforcing their sense of identity and purpose. This could be achieved through lifelong learning courses, fitness programs, or volunteer opportunities. PG refers to the ongoing development and challenges older individuals face at different life stages (22). This growth can be intellectual, emotional, social, and physical. Engaging in new learning opportunities and hobbies and overcoming daily challenges can contribute to personal achievement and fulfillment. For instance, participating in physical exercise, emotional resilience training, or friendship circles can be beneficial. As older people transition from independent living to dependent care, their difficulties may evolve due to changes in physical capabilities and social environment. AU is defined as self-assessment by personal standards (22), with this score indicating that residents maintain an acceptable level of self-determination and personal standards. Older CCRCs residents often face transitions that may challenge their autonomy, such as moving from independent living to assistance-based settings, making their psychological well-being in terms of autonomy crucial. This can be supported by involving them in care planning, providing opportunities to express preferences, and encouraging participation in community decisions.

The SF-36 (42) was utilized to evaluate the physical health of CCRC residents. The overall average physical health score of 58.33, as shown in Table 5, reflects significant physical limitations experienced by older CCRC residents that cannot be overlooked. The average scores across the four domains present a mixed picture of physical health, with strengths in physical functioning but significant challenges in areas related to daily roles, overall health, and pain management.

**Table 5:** Physical health domains ranking.

Rank	Domains	Average score (out of 100)
1	Physical functioning (PF)	71.36
2	Role functioning/physical (RF)	56.46
3	General health (GH)	53.52
4	Pain (PA)	51.97
	Average score	58.33

Specifically, physical functioning in CCRCs reveals that older residents have a relatively high level of physical ability and functionality, with a 71.36 score indicating fewer limitations in their daily activities and higher independence and mobility (37). Examples include participating in exercise classes, joining walking groups, and engaging in gardening activities.

However, three domains were identified with scores below 60, indicating significant health concerns. Role functioning/physical, which measures an individual’s ability to fulfill daily roles and activities, scored 56.46, indicating limitations residents face due to physical health constraints, such as difficulty in performing household tasks and a reduced capacity to engage in social activities. General health, which assesses health status, energy levels, and overall satisfaction, scored 53.52, indicating potential health-related issues of respondents, including issues like chronic illnesses, low energy levels, and dissatisfaction with overall health. Pain (PA), referring to unpleasant physical sensations or discomfort, was also found to be a significant concern with the lowest total score of 51.97. Physical discomfort is a major issue for CCRCs residents, with many experiencing pains that can hinder their daily activities, with chronic pain, joint pain, and fibromyalgia being prevalent (37). The survey found that 63% of respondents were 75 or older, confirming earlier findings and highlighting the importance of addressing pain management in this age group.

### 3.4 Group comparison

It has been acknowledged that the well-being of older adults may vary across different demographic variables (43). The well-being of older adults was analyzed across nine demographic variables—age, gender, marital status, total income over the past 12 months, education level, Hukou type, community location, living status, and length of stay. Significant associations with well-being were observed for both “age” and “length of stay,” with Pearson Chi-Square values of 49.172 (df = 8, *p* < 0.001) and 19.092 (df = 8, *p* = 0.014), respectively, indicating substantial variations in well-being across different age and length of stay groups.

Cross-tabulation analysis in Table 6 shows significant differences in well-being scores of age groups. As age increases, the proportion of individuals reporting high well-being diminishes. In the under-66 age group, 60.4% report high well-being, while 26.5% report it for those over 85. Medium well-being increases with age, from 36.6% in the under-66 group to 64.9% in those over 85. As individuals age, the proportion of medium well-being (50 ≤ value<75) increases, with 36.6% in the under-66 group and 64.9% in those over 85. However, the oldest group has the highest proportion of low well-being, at 8.6%. The overall trend is a decline in high well-being, an increase in medium well-being, and an upward trend in low well-being, particularly those over 85.

**Table 6:** Age and well-being crosstab analysis.

			Well-being			Total count
			25 ≤ valve<50	50 ≤ valve<75	75 ≤ valve	
Age	<66	Count	3	37	61	101
		Proportion (%)	3.0%	36.6%	60.4%	100.0%
	66–75	Count	3	103	94	200
		Proportion (%)	1.5%	51.5%	47.0%	100.0%
	76–85	Count	8	222	130	360
		Proportion (%)	2.2%	61.7%	36.1%	100.0%
	>85	Count	13	98	40	151
		Proportion (%)	8.6%	64.9%	26.5%	100.0%
Total count		Count	27	460	325	812
		Proportion (%)	3.3%	56.7%	40.0%	100.0%

A significant association between age and well-being was observed (Pearson Chi-Square = 49.172, df = 8, *p* < 0.001), indicating notable variations in well-being for different age groups.

Similarly, the significance of understanding the experiences of older individuals living in CCRCs for one to 10 years is highlighted in Table 7, with a higher proportion re-porting medium well-being (54.12%) and high well-being (40.73%). However, long-term CCRCs residents report higher levels of well-being (48.6%). In contrast, low well-being is infrequent across different lengths of stay, particularly for those residing for 3–5 years (only 0.7%). This suggests that most residents achieve at least a medium level of well-being. By contrast, most long-term CCRCs residents report medium well-being, with high well-being gradually becoming comparable in groups residing for over 10 years. Long-term residents may have increased adaptation and satisfaction with CCRCs living over time or established more stable social connections and support networks.

**Table 7:** Length of stay and well-being crosstab analysis.

			Well-being			Total
			25 ≤ valve<50	50 ≤ valve<75	75 ≤ valve	
Length of stay	0–3 year	Count	20	210	158	388
		Proportion (%)	5.15%	54.12%	40.73%	100.0%
	3–5 years	Count	2	170	103	275
		Proportion (%)	0.7%	61.8%	37.5%	100.0%
	5–10 years	Count	1	43	29	73
		Proportion (%)	1.4%	58.9%	39.7%	100.0%
	>10 years	Count	3	35	36	74
		Proportion (%)	4.1%	47.3%	48.6%	100.0%
Total		Count	26	458	326	810
		Proportion (%)	3.2%	56.5%	40.2%	100.0%

A statistically significant association was observed between length of stay and well-being (Pearson Chi-Square = 19.092, df = 8, *p* = 0.014), indicating notable variations in well-being across different length of stay groups.

## 4 Discussion

### 4.1 Well-being of older adults in CCRCs

The research findings indicate that the overall well-being of CCRCs residents is acceptable (64.24), and multiple factors influence their psychological and physical health. These influences may begin with the relocation process and include their experiences with the living environment, social interactions, and the quality of services received after moving in.

Residents’ well-being is influenced by decision-making support, stress mitigation, and engaging residents in enjoyable activities (44). Relocating to CCRCs before physical health declines can reduce stress, while those staying at home continue to face stressors that initially led them to consider relocation (45). These factors contribute to residents’ overall well-being.

CCRCs enhance the well-being of older residents by improving access to health facilities and services and providing supportive environments. Providing initiatives, such as community coordination and shuttle services, enhance residents’ sense of control, even for those with health and mobility challenges (46). Connections with surrounding amenities, such as municipal facilities, natural environments, lakes, forest trails, and reliable public transportation, further enhance residents’ well-being. Furthermore, CCRCs significantly improve the well-being of seniors by recognizing that older adults are diverse with varying interests, preferences, and life goals. They offer a combination of standard and service-oriented housing, significantly enhancing resident well-being (47). Even if not immediately used, the availability of facilities and services can enhance residents’ sense of security and well-being as seniors choose to support their well-being as they age. Moreover, health safety is prioritized through on-site medical services, providing prompt access to care and regular health assessments, especially for residents with chronic conditions or those requiring sudden medical attention (48).

CCRCs improve residents’ mental health by reducing social isolation and promoting autonomy and security. The well-being of older individuals in these communities is influenced by their social relationships and participation in activities (49–51). Social interactions within these communities reduce loneliness, enhance safety perceptions, and significantly contribute to residents’ well-being (52). Moreover, well-being is linked to independent mobility, autonomy, and control over daily life (53). CCRCs enhance residents’ well-being by ensuring safety through secure entryways, illuminated paths, and emergency alert systems. These measures make residents feel protected and secure in their environment, promoting a sense of autonomy and control over their lives (54).

The well-being of CCRCs residents is dynamic, with initial increases and declines, but ultimately higher than non-CCRCs residents (55). This may be due to initial satisfaction with CCRCs services but unsettled by diverse health problems and frequent death notices. Eventually, residents will appreciate the CCRCs as a secure place that offers independence and necessary emergency safety measures (55).

However, CCRCs can sometimes negatively impact the emotional well-being of residents, as staff can cause emotional harm. The mechanisms of these institutions often require older residents to acknowledge the significant role of management in their lives and their limited self-management capacity, leading to a decline in overall well-being (56). Furthermore, during the COVID-19 pandemic, most CCRCs residents experienced a significant decrease in their psychological well-being, including anxiety, depression, anger, and despair. Despite these challenges, CCRCs residents consistently outperformed non-CCRCs residents, highlighting the CCRCs’ ability to support their residents’ well-being, despite a global health crisis (57).

### 4.2 Group comparison

The well-being scores of different age groups within the CCRCs population offer valuable insights into aging and its impact on older people. The trend of decreasing well-being with advancing age may be due to deteriorating health conditions and loss of social connections (58). Older adults face chronic diseases, cognitive decline, and reduced physical activity, which can affect daily life quality and lead to psychological stress and emotional difficulties. Health challenges can significantly impact daily life quality, leading to psychological stress and emotional difficulties, ultimately affecting well-being (59). Older individuals may experience loss of close friends, changes in social roles post-retirement, and a shrinking social circle, resulting in loneliness and social isolation (117), ultimately reducing their overall well-being (60).

Older adults, particularly those aged 85 and above, are beneficial in promoting health-related well-being of older people, as their comprehensive support services and healthcare can effectively address the health issues these residents face (61). CCRCs provide social engagement opportunities, reducing loneliness and enhancing a sense of belonging (62). They also offer a secure environment with age-friendly facilities and emergency assistance services, ensuring a safe living environment for residents (54). These factors contribute to the overall well-being of residents. However, Liang et al. (63) suggest that refined services and care measures, such as health promotion programs, psychological support, and activities enhancing adaptability, are needed to improve the well-being of individuals aged 85 and above.

Additionally, the experiences of older adults who have lived in CCRCs for one to 10 years differ, as do their levels of well-being. The potential impact of the CCRCs living environment on the long-term well-being of residents is highlighted.

Environmental Psychology Theory (64) emphasizes the connection between environment and individual well-being, with familiarity and adaptability as key factors. Older adults in CCRCs aged 0–3 years reported high well-being, possibly due to optimistic expectations and the novelty of adjusting to a new environment (65). They may still navigate their lifestyle and establish new social connections within CCRCs (66). However, long-term CCRCs residents report significantly higher levels of well-being, as they have more time to build strong friendships and support networks within the community (9). They are also more familiar with the services and resources provided by CCRCs, allowing them to use these effectively to support their needs and hobbies (61). This suggests deep adaptation and satisfaction with the CCRCs life of long-term residents. Long-term residents may experience increased social support and belonging through participation in community activities and projects, which can enhance their well-being (67). The Social Support Theory (68) provides a theoretical basis for understanding the higher well-being reported by long-term residents.

Most CCRCs residents report satisfactory well-being, which may indicate that the living environment and services provided effectively maintain residents’ basic well-being and quality of life during adaptation (69). However, medium well-being consistently outperforms high well-being, suggesting room for improvement through increased social activities, improved healthcare services, and personalized support to meet individual needs and preferences (9). Besides, the study highlights that, while residents with longer stays generally experience increased well-being, individual factors such as health status, personality traits, and social activity participation are not negligible in impacting their well-being. Therefore, CCRCs developers should implement proactive measures tailored to residents’ unique characteristics to improve their overall well-being.

The issues of equity and scalability in CCRCs services must not be neglected. Given their market-driven nature, CCRCs predominantly attract wealthier residents. The income levels of CCRCs residents are significantly higher than Shanghai’s 2022 average of 79,610 RMB (70), with 40.6% earning between 60,000–120,000 RMB and 42.3% exceeding 120,000 RMB. Consequently, the high living and care costs in CCRCs pose substantial barriers for low-income individuals. Additionally, the strict admission criteria of some CCRCs, such as Taikang Home’s requirement for a 2 million RMB insurance fee, further limit access for lower-income groups.

Moreover, the scalability of CCRCs is another critical issue arising from their market-driven model. Given the long development durations and high initial and ongoing expenditures (4), CCRCs encounter major obstacles in broadening their reach. The lengthy return on investment cycles, coupled with strict governmental entry standards and insufficient policy support, further complicate scalability. Additionally, the complex and demanding nature of services—spanning healthcare, recreational activities, dining, and the need for highly skilled management and professional staff (71)—poses significant challenges to scalability.

Addressing these challenges requires a multifaceted approach. CCRCs developers could negotiate for tax breaks and financial subsidies to ease the burden on low-income individuals (38). Adopting a light-asset model, such as “embedded” CCRCs within existing communities, could lower construction costs. An alternative approach involves combining CCRCs services with broader social services and third-party nonprofits, leveraging existing resources to establish a comprehensive support system. Additionally, promoting long-term care insurance and incorporating smart care technologies, like telemedicine, can enhance care efficiency and reduce operational costs (54). These measures will make CCRCs more inclusive and equitable, better serving older adults from diverse socioeconomic backgrounds and ultimately improving their overall well-being.

### 4.3 Comparing well-being across developing long-term care models in China

The living arrangements significantly impact the well-being of older adults, with effects varying across different situations (4). Among older Chinese individuals, co-residential aging at home, grounded in strong intergenerational bonds and cultural norms of filial piety, is the predominant choice (72). Traditionally, the multigenerational co-residence pattern has benefited older individuals by offering material support and enhancing emotional well-being by reducing loneliness. Government legislation, such as the Elders’ Protection Law, reinforces this tradition, making older people depend on family members for comprehensive support in financial stability, physical health, and emotional well-being (73). However, this pattern has declined in the recent decade, leading to non-traditional households, such as generation-skipping and empty-nest households, influenced by urbanization-driven migration and increased financial independence (74). This shift in living arrangements has raised concerns about the well-being of older adults, particularly in terms of their emotional health and social connections. Additionally, many older Chinese individuals choose autonomous living, especially in urban areas, due to heightened economic self-sufficiency (75). The decreasing reliance on family care is further emphasized by a changing societal perception, where the traditional belief that living with children signifies a blessed old age is gradually shifting, with more older adult individuals recognizing the value of independence or alternative living arrangements.

Regarding institutional senior care in China, She-hui-fu-li-yuan (social welfare institutions) and Jing-lao-yuan (older adult respect homes) were initially established to assist financially challenged older adult individuals within communities. However, as the Chinese economy transitions to a more market-driven model, these institutions have adopted a self-financing approach, expanding their resident base to include those capable of paying for their care. Simultaneously, privately funded facilities such as Yang-lao-yuan (institutions for providing care for older adults) and CCRCs have emerged, targeting seniors with higher socio-economic status capable of covering their expenses for institutional senior living and care (76). The financial model is a key factor influencing the levels of material and emotional support provided by older adult care institutions, which is crucial to the overall well-being of residents. Residents in welfare-oriented homes often report lower well-being due to basic facilities, limited resources, inadequate care, and insufficient emotional support, whereas older adults in privately funded institutions generally experience significantly better well-being (4).

In China, choosing institutional residential older adult care is intertwined with family harmony, filial obligations, socio-cultural norms related to child-rearing and older adult care, insights into older adult care institutions, and self-assessed economic status (77). Despite a growing number of seniors leaning towards long-term care in institutions, concerns about their well-being persist. This can be attributed to such factors as stigmatization conflicting with traditional filial piety values, doubts about care quality, psychological challenges associated with moving from familiar environments, and concerns about losing connections with established social ties (78), all of which can negatively impact the emotional and mental health of older adults.

Beyond traditional home care and institutional settings, more adaptable models for senior living are emerging for China’s rapidly aging population. Particularly, as socio-economic dynamics evolve, options combining the comforts of home with the benefits of institutional care are gaining traction. Given the current circumstances, CCRCs are emerging as an alternative housing option for the growing population of older Chinese individuals (6). These facilities primarily cater to affluent older adult citizens who possess substantial disposable household income and welfare benefits, providing an environment with high-quality amenities and comprehensive care services.

When compared to traditional long-term care models, such as nursing homes and home care, CCRCs have been shown to offer advantages in terms of residents’ well-being. CCRCs residents typically experience higher levels of life satisfaction, reduced social isolation, and better overall health outcomes (79). This is partly due to the extensive resources and superior living conditions available to CCRCs residents, which naturally contribute to an elevated sense of well-being. In contrast, traditional nursing homes often focus primarily on medical care, sometimes at the expense of emotional and social needs, while home care may lack the social interaction necessary for maintaining psychological health (4).

### 4.4 Limitation and future research

While the current study provides valuable insights into the well-being of CCRCs residents, its geographic focus on Shanghai limits the generalizability of the findings to other regions in China and beyond. Future research would benefit from including geographically diverse samples to improve the external validity of the conclusions. Additionally, although the study acknowledges the importance of demographic diversity, a more in-depth exploration of demographic factors, including cultural and ethnic backgrounds, is warranted. The coexistence of 56 legally recognized ethnic groups within Chinese provinces often leads to diverse community phenomena (80). Applying the questionnaire in such diverse communities could uncover ethnic cohabitation instances and offer a more comprehensive understanding of cultural impacts.

Furthermore, the study’s reliance on self-reported data from residents may be influenced by social desirability and recall biases (81). Integrating observational and interview methods in future research could provide deeper and more nuanced interpretations. While this study employed the Ryff Scales combined with the SF-36 Health Survey for a comprehensive assessment of well-being, incorporating additional scales, such as the UCLA Loneliness Scale (82), could offer multidimensional data, capturing impacts from various domains on the well-being of older populations. The study is likewise limited by its focus on overall occupancy numbers, without detailed categorization by levels of independence. Future research should incorporate more detail to understand the proportion of survey respondents to the total eligible population.

Finally, this research primarily addresses CCRC residents and, due to time and budget constraints, has not included data from non-CCRC populations with similar demographic characteristics. Future research could include diverse living communities for older adults in China, such as community day care centers that provide daytime care and social activities, as well as retirement communities supported by government and public sector institutions offering extensive medical and living services. This approach would contribute to a more comprehensive understanding of how different living environments impact the well-being of older adults. Addressing these limitations can enhance the robustness and practical relevance of future studies.

## 5 Conclusion

The aging population in China presents challenges for older adults, making CCRCs an alternative. However, understanding the well-being of residents within these communities is limited. This study aimed to fill this gap by assessing the overall well-being of 1,107 (with 815 valid responses) residents from 13 CCRCs, covering physical and psychological dimensions across 10 domains.

The average overall well-being score of 64.24/100 indicates a satisfactory overall well-being but with significant potential for improvement. The psychological well-being score, at 70.15/100, surpasses the physical well-being score of 58.33/100, indicating a need to improve physical health to improve the overall well-being of older residents significantly. The analysis shows the positive psychological health of residents, particularly in terms of environmental mastery, self-acceptance, and positive relations. Environmental mastery scored highest, indicating adept adjustment to the CCRCs living environment. Physical health presents significant issues for older adults, but physical functioning scored relatively well, indicating the ability to undertake daily and moderately strenuous physical activities without significant hindrances. However, the residents’ Role physical, General health, and Pain scores were below 60/100, indicating daily challenges, chronic disease battles, future health concerns, and frequent or persistent physical discomfort. Furthermore, significant disparities are revealed in the well-being of older CCRCs residents based on age groups and residence length. As age increases, the proportion of people reporting high well-being decreases, particularly for those over 85. Longer residents generally have higher levels of well-being. This trend is particularly pronounced those over 85 years old. Specifically, most individuals with over a decade of residence report high well-being, while those with shorter durations generally have moderate or high levels of well-being.

## Data Availability

The raw data supporting the conclusions of this article will be made available by the authors, without undue reservation.

## References

[1] Chen L Zhang X Xu X. Health insurance and long-term care services for the disabled elderly in China: based on CHARLS data. Risk Manag Healthc Policy. (2020) 13:155–62. doi: 10.2147/RMHP.S233949 32161509 PMC7051854

[2] World Health Organization. (2024). Ageing and health in China. Available at: https://www.who.int/china/health-topics/ageing (Accessed June 30, 2024).

[3] Fang EF Xie C Schenkel JA Wu C Long Q Cui H. A research agenda for ageing in China in the 21st century: focusing on basic and translational research, long-term care, policy and social networks. Ageing Res Rev. (2020) 64:101174. doi: 10.1016/j.arr.2020.101174 32971255 PMC7505078

[4] Hu X Xia B Hu Y Skitmore M Buys L. What hinders the development of Chinese continuing care retirement community sector? A news coverage analysis. Int J Strateg Prop Manag. (2019) 23:108–16. doi: 10.3846/ijspm.2019.7436

[5] Rikard RV Berkowsky RW Cotten SR. Discontinued information and communication technology usage among older adults in continuing care retirement communities in the United States. Gerontology. (2018) 64:188–200. doi: 10.1159/000482017 29130976 PMC5828954

[6] Wang X. Xia B. Burton L. O. (2022). An approach to construct Bayesian networks for use in living environment field, taking Chinese CCRCs as the example. *In* Proceedings of the 7th New Zealand built environment research symposium (NZBERS): Creating capacity and capability for the future of the built environment (pp. 277–293). Massey University.

[7] Shinan-Altman S Gum AM Ayalon L. Moving to a continuing care retirement community or staying in the community? A comparison between American and Israeli older adults. J Appl Gerontol. (2020) 39:1221–9. doi: 10.1177/0733464819879015 31587605

[8] Medvedev ON Landhuis CE. Exploring constructs of well-being, happiness and quality of life. PeerJ. (2018) 6:e4903. doi: 10.7717/peerj.4903 29876148 PMC5985772

[9] Engelen L Rahmann M de Jong E. Design for healthy ageing–the relationship between design, well-being, and quality of life: a review. Build Res Inform. (2022) 50:19–35. doi: 10.1080/09613218.2021.1984867

[10] Vanleerberghe P De Witte N Claes C Schalock RL Verté D. The quality of life of older people aging in place: a literature review. Qual Life Res. (2017) 26:2899–907. doi: 10.1007/s11136-017-1651-0 28707047

[11] Zhang Z Zhang J. Perceived residential environment of neighborhood and subjective well-being among the elderly in China: a mediating role of sense of community. J Environ Psychol. (2017) 51:82–94. doi: 10.1016/j.jenvp.2017.03.004

[12] Zubrick SR Kovess-Masfety V. Chapter 12: Indicators of mental health. In: H Herrman S Saxena R Moody, editors. Promoting mental health: Concepts, emerging evidence, practice. World Health Organisation. (2005) 148–168.

[13] Bradburn NM. The structure of psychological well-being. Chicago: ALDINE publishing company (1969).

[14] Schimmack U Diener E. Predictive validity of explicit and implicit self-esteem for subjective well-being. J Res Pers. (2003) 37:100–6. doi: 10.1016/S0092-6566(02)00532-9

[15] Radloff LS. The CES-D scale: a self-report depression scale for research in the general population. Appl Psychol Meas. (1977) 1:385–401. doi: 10.1177/01466216770010030

[16] Laidlaw K Baikie E. Psychotherapy and demographic change. *Nordic*. Psychology. (2007) 59:45–58. doi: 10.1027/1901-2276.59.1.45

[17] Diener ED Emmons RA Larsen RJ Griffin S. The satisfaction with life scale. J Pers Assess. (1985) 49:71–5. doi: 10.1207/s15327752jpa4901_13 16367493

[18] Waterman AS Schwartz SJ Zamboanga BL Ravert RD Williams MK Bede Agocha V. The questionnaire for Eudaimonic well-being: psychometric properties, demographic comparisons, and evidence of validity. J Posit Psychol. (2010) 5:41–61. doi: 10.1080/17439760903435208 34326891 PMC8317967

[19] Woźniak B Tobiasz-Adamczyk B. Quality of life and well-being. Krakow: Jagiellonian University (2014).

[20] Streiner DL Norman GR Cairney J. Health measurement scales: A practical guide to their development and use. New York: Oxford University Press (2015).

[21] Cooke PJ Melchert TP Connor K. Measuring well-being: a review of instruments. Couns Psychol. (2016) 44:730–57. doi: 10.1177/001100001663350

[22] Ryff CD. Beyond Ponce de Leon and life satisfaction: new directions in quest of successful ageing. Int J Behav Dev. (1989) 12:35–55. doi: 10.1177/016502548901200102

[23] Frisch MB Cornell J Villanueva M Retzlaff PJ. Clinical validation of the quality of life inventory. A measure of life satisfaction for use in treatment planning and outcome assessment. Psychol Assess. (1992) 4:92. doi: 10.1037/1040-3590.4.1.92

[24] Lent RW. Toward a unifying theoretical and practical perspective on well-being and psychosocial adjustment. J Couns Psychol. (2004) 51:482. doi: 10.1037/0022-0167.51.4.482

[25] Palombi BJ. Psychometric properties of wellness instruments. J Couns Dev. (1992) 71:221–5. doi: 10.1002/j.1556-6676.1992.tb02204.x

[26] Vanhoutte B. The multidimensional structure of subjective well-being in later life. J Popul Age. (2014) 7:1–20. doi: 10.1007/s12062-014-9092-9 PMC4102758 25089162

[27] Nieboer A Lindenberg S Boomsma A Bruggen ACV. Dimensions of well-being and their measurement: the SPF-IL scale. Soc Indic Res. (2005) 73:313–53. doi: 10.1007/s11205-004-0988-2

[28] Patrick CJ Curtin JJ Tellegen A. Development and validation of a brief form of the multidimensional personality questionnaire. Psychol Assess. (2002) 14:150. doi: 10.1037/1040-3590.14.2.150 12056077

[29] Mezzich JE Cohen NL Ruiperez MA Banzato CE Zapata-Vega MI. The multicultural quality of life index: presentation and validation. J Eval Clin Pract. (2011) 17:357–64. doi: 10.1111/j.1365-2753.2010.01609.x 21208350

[30] Neugarten BL Havighurst RJ Tobin SS. The measurement of life satisfaction. J Gerontol. (1961) 16:134–43. doi: 10.1093/geronj/16.2.134 13728508

[31] Taft C Karlsson J Sullivan M. Performance of the Swedish SF-36 version 2.0. Qual Life Res. (2004) 13:251–6. doi: 10.1023/B:QURE.0000015290.76254.a5 15058805

[32] Ryff CD Keyes CLM. The structure of psychological well-being revisited. J Pers Soc Psychol. (1995) 69:719. doi: 10.1037//0022-3514.69.4.719 7473027

[33] Kim K Lehning AJ Sacco P. Assessing the factor structure of well-being in older adults: findings from the National Health and aging trends study. Aging Ment Health. (2016) 20:814–22. doi: 10.1080/13607863.2015.1037245 25915703

[34] Abbott RA Ploubidis GB Huppert FA Kuh D Wadsworth ME Croudace TJ. Psychometric evaluation and predictive validity of Ryff’s psychological well-being items in a UK birth cohort sample of women. Health Qual Life Outcomes. (2006) 4:1–16. doi: 10.1186/1477-7525-4-76 17020614 PMC1634744

[35] Pedhazur EJ Schmelkin LP. Measurement, design, and analysis: an integrated approach. London: Psychology Press (2013).

[36] Van Dierendonck D Lam H. Interventions to enhance eudaemonic psychological well-being: a meta-analytic review with Ryff’s scales of psychological well-being. Appl Psychol Health Well Being. (2023) 15:594–610. doi: 10.1111/aphw.12398 36073601

[37] Ware JE. SF-36 health survey update. Spine. (2000) 25:3130–9. 11124729 10.1097/00007632-200012150-00008

[38] Lam TY Yan J. Continuing care retirement community senior housing in Shanghai: an analysis of the development barriers. Int J Hous Markets Anal. (2022) 15:780–99. doi: 10.1108/ijhma-04-2021-0038

[39] Little RJ Rubin DB. Statistical analysis with missing data. New Jersey: John Wiley & Sons (2019).

[40] Lee CT Leoutsakos JM Lyketsos CG Steffens DC Breitner JC Norton MC. Latent class-derived subgroups of depressive symptoms in a community sample of older adults: the Cache County study. Int J Geriatr Psychiatry. (2012) 27:1061–9. doi: 10.1002/gps.2824 22135008 PMC3419796

[41] Chinanews. (2022). Resident income rankings for 2021 announced. Available at: http://finance.people.com.cn/n1/2022/0120/c1004-32335731.html (Accessed June 30, 2024).

[42] Ware JE Kosinski M Gandek B Aaronson NK Apolone G Bech P. The factor structure of the SF-36 health survey in 10 countries: results from the IQOLA project. J Clin Epidemiol. (1998) 51:1159–65. doi: 10.1016/S0895-4356(98)00107-3 9817133

[43] Papageorge NW Zahn MV Belot M Van den Broek-Altenburg E Choi S Jamison JC. Socio-demographic factors associated with self-protecting behavior during the Covid-19 pandemic. J Popul Econ. (2021) 34:691–738. doi: 10.1007/s00148-020-00818-x 33462529 PMC7807230

[44] Cooper AM Lim YJ. SilverComm: Marketing practices and messages for the age of aging. London: Rowman & Littlefield (2023).

[45] Heisler E Evans GW Moen P. Health and social outcomes of moving to a continuing care retirement community. J Hous Elder. (2003) 18:5–23. doi: 10.1300/J081v18n01_02

[46] Kochtitzky CS Freeland AL Yen IH. Ensuring mobility-supporting environments for an aging population: critical actors and collaborations. J Aging Res. (2011) 2011:138931. doi: 10.4061/2011/138931 21766029 PMC3134094

[47] Ayalon L Avidor S. ‘We have become prisoners of our own age’: from a continuing care retirement community to a total institution in the midst of the COVID-19 outbreak. Age Ageing. (2021) 50:664–7. doi: 10.1093/ageing/afab013 33951154 PMC7929415

[48] Kuch LD. Educating residents of a continuing care retirement community to make intentional care transitions. Pennsylvania: Lancaster Bible College (2020).

[49] Philip KE Polkey MI Hopkinson NS Steptoe A Fancourt D. Social isolation, loneliness and physical performance in older-adults: fixed effects analyses of a cohort study. Sci Rep. (2020) 10:13908. doi: 10.1038/s41598-020-70483-3 32807857 PMC7431531

[50] Asante S Karikari G. Social relationships and the health of older adults: an examination of social connectedness and perceived social support. J Age Longev. (2022) 2:49–62. doi: 10.3390/jal2010005

[51] Liu JE Tian JY Yue P Wang YL Du XP Chen SQ. Living experience and care needs of Chinese empty-nest elderly people in urban communities in Beijing, China: a qualitative study. Int J Nurs Sci. (2015) 2:15–22. doi: 10.1016/j.ijnss.2015.01.008

[52] Sen K Prybutok G Prybutok V. The use of digital technology for social well-being reduces social isolation in older adults: a systematic review. SSM Popul Health. (2022) 17:101020. doi: 10.1016/j.ssmph.2021.101020 35024424 PMC8733322

[53] Tao Y Petrović A van Ham M. Working from home and subjective wellbeing during the COVID-19 pandemic: the role of pre-COVID-19 commuting distance and mode choices. J Transp Geogr. (2023) 112:103690. doi: 10.1016/j.jtrangeo.2023.103690

[54] Kumar S Underwood SH Masters JL Manley NA Konstantzos I Lau J. Ten questions concerning smart and healthy built environments for older adults. Build Environ. (2023) 244:110720. doi: 10.1016/j.buildenv.2023.110720

[55] Smith EK. Residential satisfaction, psychological well-being, and personality traits: Effects on relocation among older adults. Kansas: University of Kansas (2014).

[56] Oswald F Rowles GD. Beyond the relocation trauma in old age: new trends in elders’ residential decisions In: Hans-Werner W Tesch-Romer C Hoff A Hendricks J, editors. New dynamics in old age. London: Routledge (2017). 127–52.

[57] Green O Ayalon L. “Home is where my couch is”: the role of possessions in the process of moving and adjusting to continuing care retirement communities. Qual Health Res. (2019) 29:577–88. doi: 10.1177/1049732318780350 29947582

[58] Velaithan V Tan MM Yu TF Liem A Teh PL Su TT. The association of self-perception of aging and quality of life in older adults: a systematic review. The Gerontologist. (2023) 64:gnad041. doi: 10.1093/geront/gnad041 PMC10943510 37029753

[59] Izquierdo M Duque G Morley JE. Physical activity guidelines for older people: knowledge gaps and future directions. Lancet Healthy Longev. (2021) 2:e380–3. doi: 10.1016/S2666-7568(21)00079-9 36098146

[60] Kauppi M Virtanen M Pentti J Aalto V Kivimäki M Vahtera J. Social network ties before and after retirement: a cohort study. Eur J Ageing. (2021) 18:503–12. doi: 10.1007/s10433-021-00604-y 34786012 PMC8563893

[61] Chaulagain S Li J Pizam A. What matters, and what matters most? Exploring resident satisfaction in continuing care retirement communities. Int J Contemp Hosp Manag. (2022) 34:2472–95. doi: 10.1108/IJCHM-09-2021-1105

[62] Boeder J Hwang S Chan T. Engagement with life among the oldest-old in assisted living facilities: enriching activities and developmental adaptation to physical loss. Age Soc. (2022) 42:1191–212. doi: 10.1017/S0144686X20001488

[63] Liang Y Shang S Gao Y Zhai J Cheng X Yang C. Measurements of intrinsic capacity in older adults: a scoping review and quality assessment. J Am Med Dir Assoc. (2023) 24:267–76. doi: 10.1016/j.jamda.2022.09.011 36332688

[64] Mehrabian A Russell JA. A measure of arousal seeking tendency. Environ Behav. (1973) 5:315.

[65] Oishi S Diener E Lucas RE. The optimum level of well-being: can people be too happy? Perspect Psychol Sci. (2007) 2:346–60. doi: 10.1111/j.1745-6916.2007.00048.x 26151972

[66] Jolanki O Vilkko A. The meaning of a “sense of community” in a Finnish senior co-housing community. J Hous Elder. (2015) 29:111–25. doi: 10.1080/02763893.2015.989767

[67] Sugihara S Evans GW. Place attachment and social support at continuing care retirement communities. Environ Behav. (2000) 32:400–9. doi: 10.1177/0013916002197258

[68] Cohen S Wills TA. Stress, social support, and the buffering hypothesis. Psychol Bull. (1985) 98:310. doi: 10.1037/0033-2909.98.2.310 3901065

[69] Ayalon L. Quality of life of older adults in continuing care retirement communities In: Sirgy MJ, editor. Handbook of Qualityof life research. Cheltenham: Edward Elgar Publishing (2024). 339–54.

[70] Shanghai Municipal Bureau of Statistics. (2023). The 2022 per capita disposable income and consumption expenditure of residents. Available at: https://tjj.sh.gov.cn/ydsj71/20230118/5d288f12efbc4c9298d7babbf1b1b7a7.html (Accessed July 26, 2024).

[71] Kabadayi S Hu K Lee Y Hanks L Walsman M Dobrzykowski D. Fostering older adult care experiences to maximize well-being outcomes: a conceptual framework. J Serv Manag. (2020) 31:953–77. doi: 10.1108/josm-11-2019-0346

[72] Qiu FX Zhan HJ Liu J Barrett PM. Downward transfer of support and care: understanding the cultural lag in rural China. Age Soc. (2022) 42:1422–47. doi: 10.1017/S0144686X2000152X

[73] Qi X. Filial obligation in contemporary China: evolution of the culture-system. J Theory Soc Behav. (2015) 45:141–61. doi: 10.1111/jtsb.12052

[74] Su Z Hu Z Peng X. The impact of changes in China’s family patterns on family pension functions. Int J Health Plann Manag. (2017) 32:351–62. doi: 10.1002/hpm.2436 28736874

[75] Fu YY Chui EWT. Determinants of patterns of need for home and community-based care services among community-dwelling older people in urban China: the role of living arrangement and filial piety. J Appl Gerontol. (2020) 39:712–21. doi: 10.1177/0733464819871875 32517576

[76] Li Y Wang C Wong H. The role of the private sector in China’s senior care industry. Options Aged Care China. (2018) 155–189. doi: 10.1596/978-1-4648-1075-6_ch4

[77] Xing Y Pei R Qu J Wang J Zhou H Wang Z. Urban-rural differences in factors associated with willingness to receive eldercare among the elderly: a cross-sectional survey in China. BMJ Open. (2018) 8:e020225. doi: 10.1136/bmjopen-2017-020225 PMC5988108 29858413

[78] Wang Z Xing Y Yan W Sun X Zhang X Huang S. Effects of individual, family and community factors on the willingness of institutional elder care: a cross-sectional survey of the elderly in China. BMJ Open. (2020) 10:e032478. doi: 10.1136/bmjopen-2019-032478 PMC7044895 32075825

[79] Zhang M Pan Y. Design of Sustainable Senior-Friendly Community Transportation Services. Sustain For. (2021) 13:13078. doi: 10.3390/su132313078

[80] Yao J Yan X Liu S. Linguistic landscape in Liangshan Yi autonomous prefecture: the case of an ethnic minority region in China. Int J Multiling. (2023) 20:169–88. doi: 10.1080/14790718.2020.1800018

[81] DeMaio TJ. Social desirability and survey In: Turner CF Martin E, editors. Surveying Subjective Phenomena. New York, NY: Russell Sage Foundation (1984). 257–81.

[82] Russell DW. UCLA loneliness scale (version 3): reliability, validity, and factor structure. J Pers Assess. (1996) 66:20–40. doi: 10.1207/s15327752jpa6601_2 8576833

[83] Lawton MP. The Philadelphia geriatric center morale scale: A revision. J Gerontol. (1975) 30:85–9. doi: 10.1093/geronj/30.1.85 1109399

[84] Dupuy HJ. The general well-being schedule. In: I McDowell C Newell, editors. Measuring health: A guide to rating scales and questionnaire, (2nd edn). USA: Oxford University Press (1977) 206–213.

[85] Rosenberg M. Rosenberg self-esteem scale. APA PsycTests. (1965) 61:18. doi: 10.1037/t01038-000

[86] Ferrans CE Powers MJ. Quality of life index: development and psychometric properties. Adv Nurs Sci. (1985) 8:15–24. doi: 10.1097/00012272-198510000-00005 3933411

[87] Csikszentmihalyi M Larson R. Validity and reliability of the experience-sampling method. J Nerv Ment Dis. (1987) 175:526–36. doi: 10.1097/00005053-198709000-00004 3655778

[88] Antonovsky A. Unraveling the mystery of health: How people manage stress and stay well. San Francisco: Jossey-Bass (1987).

[89] Watson D Clark LA Tellegen A. Development and validation of brief measures of positive and negative affect: the PANAS scales. J Pers Soc Psychol. (1988) 54:1063. doi: 10.1037/0022-3514.54.6.1063 3397865

[90] EuroQOl Group. (1990). EQ-5D-3L. Available at: https://euroqol.org/information-and-support/euroqol-instruments/eq-5d-3l/ (Accessed Sep 22, 2024).

[91] Tibblin G Tibblin B Peciva S Kullman S Svärdsudd K. The Göteborg quality of life instrument-an assessment of well-being and symptoms among men born 1913 and 1923. Scand J Prim Health Care. (1990) 1:33–8. 2100362

[92] Ware JE Sherbourne CD. The MOS 36-ltem short-form health survey (SF-36): I. Conceptual framework and item selection. Med Care. (1992) 30:473–83. doi: 10.1097/00005650-199206000-00002 1593914

[93] Bech P Olsen LR Kjoller M Rasmussen NK. Measuring well-being rather than the absence of distress symptoms: a comparison of the SF-36 mental health subscale and the WHO-five well-being scale. Int J Methods Psychiatr Res. (2003) 12:85–91. doi: 10.1002/mpr.145 12830302 PMC6878541

[94] Ruta DA Garratt AM Leng M Russell IT MacDonald LM. A new approach to the measurement of quality of life: the patient-generated index. Med Care. (1994) 32:1109–26. doi: 10.1097/00005650-199411000-00004 7967852

[95] Scheier MF Carver CS Bridges MW. Distinguishing optimism from neuroticism (and trait anxiety, self-mastery, and self-esteem): a reevaluation of the life orientation test. J Pers Soc Psychol. (1994) 67:1063. doi: 10.1037//0022-3514.67.6.1063 7815302

[96] Watson D Clark LA. Mood and anxiety symptom questionnaire. J Behav Ther Exp Psychiatry. (1991). doi: 10.1037/t13679-000

[97] Cummins RA. The domains of life satisfaction: an attempt to order chaos. Soc Indic Res. (1996) 38:303–28. doi: 10.1007/BF00292050

[98] Kaplan RM Sieber WJ Ganiats TG. The quality of well-being scale: comparison of the interviewer-administered version with a self-administered questionnaire. Psychol Health. (1997) 12:783–91. doi: 10.1080/08870449708406739

[99] Whoqol Group. Development of the World Health Organization WHOQOL-BREF quality of life assessment. Psychol Med. (1998) 28:551–8. doi: 10.1017/S0033291798006667 9626712

[100] Prince MJ Reischies F Beekman AT Fuhrer R Jonker C Kivela SL. Development of the EURO–D scale–a European Union initiative to compare symptoms of depression in 14 European centres. Br J Psychiatry. (1999) 174:330–8. doi: 10.1192/bjp.174.4.330 10533552

[101] Goldberg D Williams P. General health questionnaire (GHQ). Swindon: NFER-Nelson (2000).

[102] Hyde M Wiggins RD Higgs P Blane DB. A measure of quality of life in early old age: the theory, development and properties of a needs satisfaction model (CASP-19). Aging Ment Health. (2003) 7:186–94. doi: 10.1080/1360786031000101157 12775399

[103] Ventegodt S Merrick J Andersen NJ. Measurement of quality of life III. From the IQOL theory to the global, generic SEQOL questionnaire. Sci World J. (2003) 3:972–91. doi: 10.1100/tsw.2003.77 PMC5974868 14570988

[104] Kahneman D Krueger AB Schkade DA Schwarz N Stone AA. A survey method for characterizing daily life experience: the day reconstruction method. Science. (2004) 306:1776–80. doi: 10.1126/science.110357 15576620

[105] Ingersoll-Dayton B Krause N. Self-forgiveness: a component of mental health in later life. Res Aging. (2005) 27:267–89. doi: 10.1177/0164027504274122

[106] Peterson C Park N Seligman ME. Orientations to happiness and life satisfaction: the full life versus the empty life. J Happiness Stud. (2005) 6:25–41. doi: 10.1007/s10902-004-1278-z

[107] Al-Naser F Ridha HMA Figley CR. Adopting a strength-based, indigenous-focused approach to studying Kuwait post-traumatic stress. Digest Middle East Stud. (2005) 14:1–10. doi: 10.1111/j.1949-3606.2005.tb00893.x

[108] Tennant R Hiller L Fishwick R Platt S Joseph S Weich S. The Warwick-Edinburgh mental well-being scale (WEMWBS): development and UK validation. Health Qual Life Outcomes. (2007) 5:1–13. doi: 10.1186/1477-7525-5-63 18042300 PMC2222612

[109] Katerndahl D Oyiriaru D. Assessing the biopsychosociospiritual model in primary care: development of the biopsychosociospiritual inventory (BioPSSI). Int J Psychiatry Med. (2007) 37:393–414. doi: 10.2190/PM.37 18441628

[110] Rothmann S Ekkerd J. The validation of the perceived wellness survey in the south African police service. SA J Ind Psychol. (2007) 33:35–42. doi: 10.4102/sajip.v33i3.393

[111] Kahneman D Deaton A. High income improves evaluation of life but not emotional well-being. Proc Natl Acad Sci. (2010) 107:16489–93. doi: 10.1073/pnas.1011492107 20823223 PMC2944762

[112] Bringsén Å Andersson HI Ejlertsson G. Development and quality analysis of the Salutogenic health indicator scale (SHIS). Scand J Public Health. (2009) 37:13–9. doi: 10.1177/140349480809891 19141550

[113] Vaingankar JA Subramaniam M Chong SA Abdin E Orlando Edelen M Picco L. The positive mental health instrument: development and validation of a culturally relevant scale in a multi-ethnic Asian population. Health Qual Life Outcomes. (2011) 9:1–18. doi: 10.1186/1477-7525-9-92 22040157 PMC3229450

[114] Kinderman P Schwannauer M Pontin E Tai S. The development and validation of a general measure of well-being: the BBC well-being scale. Qual Life Res. (2011) 20:1035–42. doi: 10.1007/s11136-010-9841-z 21243528

[115] Bann CM Kobau R Lewis MA Zack MM Luncheon C Thompson WW. Development and psychometric evaluation of the public health surveillance well-being scale. Qual Life Res. (2012) 21:1031–43. doi: 10.1007/s11136-011-0002-9 21947657

[116] Supranowicz P Paź M. Holistic measurement of well-being: psychometric properties of the physical, mental and social well-being scale (PMSW-21) for adults. Rocz Panstw Zakl Hig. (2014) 65:251–8. 25247806

[117] Hu J Fitzgerald SM Owen AJ Ryan J Joyce J Chowdhury E. Social isolation, social support, loneliness and cardiovascular disease risk factors: a cross-sectional study among older adults. Int J Geriatr Psychiatry. (2021) 36:1795–809. doi: 10.1002/gps.5601 34231940

[118] Wang M Wu J Yan H. The effect of horticultural therapy on older adults in pension institutions: a systematic review. Geriatr Nurs. (2023) 51:25–32. doi: 10.1016/j.gerinurse.2023.02.006 36878128

[119] Ryff CD. (1995). Psychological well-being in adult life. Curr Dir Psychol Sci. 4:99–104. doi: 10.1111/1467-8721.ep1077239

[120] Kozma A Stones MJ. (1980). The measurement of happiness: Development of the Memorial University of Newfoundland Scale of Happiness (MUNSH). J Gerontol. 35:906–912. doi: 10.1093/geronj/35.6.906 7440930

[121] Eysenck MW. (1985). Anxiety and cognitive-task performance. Pers Individ Dif. 6:579–586. doi: 10.1016/0191-8869(85)90007-8

