# Supplementary material for: Understanding the well-being of residents in Chinese Continuing Care Retirement Communities—a case of Shanghai
Source: Front Public Health. 2024 Oct 4;12:1457022. doi: 10.3389/fpubh.2024.1457022 (PMC11486756; doi:10.3389/fpubh.2024.1457022)
Supplement: Supplementary file 1 [file Data_Sheet_1.docx]

**Appendix A Questionnaire**

Dear madam/sir:

Greeting!

We are currently conducting field surveys in your community, in collaboration with Queensland University of Technology (QUT) and the China Scholarship Council (CSC), to investigate the impact of living environment factors on the well-being of CCRCs residents in China. The survey is designed to gather comprehensive information and consists of a total of 84 anonymous questions. It includes sections on social demographics (9 questions), the Ryff 54-item psychological well-being scale (54 questions), and the physical health section of the 36-item short-form survey (SF-36) 2.0 Version (21 questions). Your participation in this survey is greatly appreciated. As a token of our appreciation, we will provide a RMB 20 supermarket voucher as a reward for completing the questionnaire.

**PART I Socio-demographics（9）**

Guidance: The following questions are Socio-demographics questions. There are 9 questions in total. Please tick the matching options – “✔”.

For example: if you are 65 years old, please tick 56-65.

| E.g., | Age | ≤55 | ✔56-65 | 66-75 | 76-85 | ＞85 |
| --- | --- | --- | --- | --- | --- | --- |

| PART I. SOCIO-DEMOGRAPHICS | | 1 | 2 | 3 | 4 | 5 |
| --- | --- | --- | --- | --- | --- | --- |
| SOCIO-DEMOGRAPHICS | | | | | | |
| 1 | Age | ≤55 | 56-65 | 66-75 | 76-85 | ＞85 |
| 2 | Gender | Male | Female |  |  |  |
| 3 | Marital status | Unmarried | Married | Divorced | Widowed |  |
| 4 | Annual total income in 2020 (wage income and other sources of income, such as rent, welfare, social security, pension, stocks, bonds, interest on savings, etc.). | ≤60,000 RMB | 60,001-120,000 RMB | 120,001-180,000 RMB | 180,001-240,000 RMB | > 240,001 RMB |
| 5 | Education level | Junior high school and below | High school or technical secondary school | Specialist qualifications | Bachelor | Master and above |
| 6 | Migration and hukou status | Urban residents | Cross-county city-to-urban migration | Cross-county rural-to-urban migration | County-level rural-to-urban migration | In-situ urbanization of rural residents |
| 7 | Years of residence | ≤1 year | 1-2 years | 2-3 years | 3-4 years | ＞4 years |
| 8 | Residence diversity | Bungalow | House | Apartment | Dormitory | Others |
| 9 | Location | Urban | Suburban | Rural |  |  |

**PART II. Ryff Scales of Psychological Well-Being-54 Items（54）**

Guidance: This section contains 54 questions that assess your feelings about yourself and your life. Please choose the option that best reflects your true feelings by selecting the corresponding box (“✔”). There are no right or wrong answers. You will be using a five-point scoring method, where 1 represents “strongly disagree,” 2 represents “disagree,” 3 represents “slightly disagree,” 4 represents “slightly agree,” 5 represents “agree,” and 6 represents “strongly agree.”

For example: If you strongly disagree with the statement “My decisions are not usually influenced by what everyone else is doing,” please tick “1.”

| E.g., | My decisions are not usually influenced by what everyone else is doing | ✔1 Strongly disagree | 2 Disagree | 3 A little disagree | 4 A little agree | 5 Agree | 6 Strongly agree |
| --- | --- | --- | --- | --- | --- | --- | --- |

| PART II. RYFF SCALES OF PSYCHOLOGICAL WELL-BEING-54 ITEMS | | 1 | 2 | 3 | 4 | 5 | 6 |
| --- | --- | --- | --- | --- | --- | --- | --- |
| AUTONOMY | | | | | | | |
| 1 | My decisions are not usually influenced by what everyone else is doing |  |  |  |  |  |  |
| 2 | I have confidence in my opinions even if they are contrary to the general consensus |  |  |  |  |  |  |
| 3 | I tend to worry about what other people think of me |  |  |  |  |  |  |
| 4 | I often change my mind about decisions if my friends or family disagree |  |  |  |  |  |  |
| 5 | I am not afraid to voice my opinions, even when they are in opposition to the opinions of most people |  |  |  |  |  |  |
| 6 | Being happy with myself is more important to me than having others approve of me |  |  |  |  |  |  |
| 7 | It’s difficult for me to voice my opinions on controversial matters |  |  |  |  |  |  |
| 8 | I tend to be influenced by people with strong opinions |  |  |  |  |  |  |
| 9 | I judge myself by what I think is important, not by what others think is important |  |  |  |  |  |  |
| ENVIRONMENTAL MASTERY | | | | | | | |
| 10 | I am good at juggling my time so that I can fit everything in that needs to get done |  |  |  |  |  |  |
| 11 | I often feel overwhelmed by my responsibilities |  |  |  |  |  |  |
| 12 | I am quite good at managing aging the many responsibilities of my daily life |  |  |  |  |  |  |
| 13 | I do not fit very well with the people and community around me |  |  |  |  |  |  |
| 14 | I have difficulty arranging my life in a way that is satisfying to me |  |  |  |  |  |  |
| 15 | I have been able to create a lifestyle for myself that is much to my liking |  |  |  |  |  |  |
| 16 | I generally do a good job of taking care of my personal finances and affairs |  |  |  |  |  |  |
| 17 | In general, I feel I am in charge of the situation in which I live |  |  |  |  |  |  |
| 18 | The demands of everyday life often get me down |  |  |  |  |  |  |
| PERSONAL GROWTH | | | | | | | |
| 19 | I am not interested in activities that will expand my horizons |  |  |  |  |  |  |
| 20 | I have the sense that I have developed a lot as a person over time |  |  |  |  |  |  |
| 21 | When I think about it, I haven’t really improved much as a person over the years |  |  |  |  |  |  |
| 22 | I think it is important to have new experiences that challenge how I think about myself and the world |  |  |  |  |  |  |
| 23 | I don’t want to try new ways of doing things – my life is fine the way it is |  |  |  |  |  |  |
| 24 | I do not enjoy being in new situations that require me to change my old familiar ways of doing things |  |  |  |  |  |  |
| 25 | There is truth to the saying you can’t teach an old dog new tricks |  |  |  |  |  |  |
| 26 | For me, life has been a continuous process of learning, changing, and growing |  |  |  |  |  |  |
| 27 | I gave up trying to make big improvements or changes in my life a long time ago |  |  |  |  |  |  |
| POSITIVE RELATIONS WITH OTHERS | | | | | | | |
| 28 | I don’t have many people who want to listen when I need to talk |  |  |  |  |  |  |
| 29 | I enjoy personal and mutual conversations with family members and friends |  |  |  |  |  |  |
| 30 | I often feel lonely because I have few close friends with whom to share my concerns |  |  |  |  |  |  |
| 31 | It seems to me that most other people have more friends than I do |  |  |  |  |  |  |
| 32 | People would describe me as a giving person, willing to share my time with others |  |  |  |  |  |  |
| 33 | Most people see me as loving and affectionate |  |  |  |  |  |  |
| 34 | I know I can trust my friends, and they know they can trust me |  |  |  |  |  |  |
| 35 | Maintaining close relationships has been difficult and frustrating for me |  |  |  |  |  |  |
| 36 | I have not experienced many warm and trusting relationships with others |  |  |  |  |  |  |
| PURPOSE IN LIFE | | | | | | | |
| 37 | I enjoy making plans for the future and working to make them a reality |  |  |  |  |  |  |
| 38 | My daily activities often seem trivial and unimportant to me |  |  |  |  |  |  |
| 39 | I am an active person in carrying out the plans I set for myself |  |  |  |  |  |  |
| 40 | I tend to focus on the present because the future nearly always brings me problems |  |  |  |  |  |  |
| 41 | I don’t have a good sense of what it is I am trying to accomplish in life |  |  |  |  |  |  |
| 42 | I sometimes feel as if I have done all there is to do in life |  |  |  |  |  |  |
| 43 | I used to set goals for myself, but that now seems like a waste of time |  |  |  |  |  |  |
| 44 | Some people wander aimlessly through life, but I am not one of them |  |  |  |  |  |  |
| 45 | I live life one day at a time and don’t really think about the future |  |  |  |  |  |  |
| SELF-ACCEPTANCE | | | | | | | |
| 46 | I feel like many of the people I know have gotten more out of life than I have |  |  |  |  |  |  |
| 47 | In general, I feel confident and positive about myself |  |  |  |  |  |  |
| 48 | When I compare myself to friends and acquaintances, it makes me feel good about who I am |  |  |  |  |  |  |
| 49 | My attitude about myself is probably not as positive as most people feel about themselves |  |  |  |  |  |  |
| 50 | I made some mistakes in the past, but I feel that all in all everything has worked out for the best |  |  |  |  |  |  |
| 51 | The past had its ups and downs, but in general, I wouldn’t want to change it |  |  |  |  |  |  |
| 52 | In many ways, I feel disappointed about my achievements in life |  |  |  |  |  |  |
| 53 | When I look at the story of my life, I am pleased with how things have turned out |  |  |  |  |  |  |
| 54 | I like most parts of my personality |  |  |  |  |  |  |

**PART III. 36-Item Short Form Survey (5)**

Guidance: This section pertains to your perceptions of your health. Your responses will help us understand how you feel and how well you are able to perform your usual activities. Please select only one answer for each question. There are a total of 21 questions. Please tick the corresponding box (“✔”). If you are unsure about how to answer a question, please provide the best answer you can.

For example: Regarding “Moderate activities, such as moving a table, pushing a vacuum cleaner, bowling, or playing golf,” if you feel that your health significantly limits you, please tick “1.”

| E.g., | Moderate activities, such as moving a table, pushing a vacuum cleaner, bowling, or playing golf | ✔ Yes, limited a lot | Yes, limited a little | No, not limited at all |  |  |
| --- | --- | --- | --- | --- | --- | --- |

| PART III. 36-ITEM SHORT FORM SURVEY | | 1 | 2 | 3 | 4 | 5 |
| --- | --- | --- | --- | --- | --- | --- |
| PHYSICAL FUNCTIONING (PF) 2+8  The following two questions are about activities you might do during a typical day. Does your health now limit you in these activities? If so, how much? | | | | | | |
| 1 | Moderate activities, such as moving a table, pushing a vacuum cleaner, bowling, or playing golf | Yes, limited a lot | Yes, limited a little | No, not limited at all |  |  |
| 2 | Climbing several flights of stairs | Yes, limited a lot | Yes, limited a little | No, not limited at all |  |  |
| 3 | Moderate activities, such as moving a table, pushing a vacuum cleaner, bowling or playing golf | Yes, limited a lot | Yes, limited a little | No, not limited at all |  |  |
| 4 | Lifting or carrying groceries | Yes, limited a lot | Yes, limited a little | No, not limited at all |  |  |
| 5 | Climbing one flight of stairs | Yes, limited a lot | Yes, limited a little | No, not limited at all |  |  |
| 6 | Bending, kneeling, or stooping | Yes, limited a lot | Yes, limited a little | No, not limited at all |  |  |
| 7 | Walking more than a mile | Yes, limited a lot | Yes, limited a little | No, not limited at all |  |  |
| 8 | Walking several blocks | Yes, limited a lot | Yes, limited a little | No, not limited at all |  |  |
| 9 | Walking one block | Yes, limited a lot | Yes, limited a little | No, not limited at all |  |  |
| 10 | Bathing or dressing yourself | Yes, limited a lot | Yes, limited a little | No, not limited at all |  |  |
| ROLE-PHYSICAL (RP) 2+2  During the past 4 weeks, have you had any of the following problems with your work or other regular activities as a result of your physical health? | | | | | | |
| 11 | Accomplished less than you would like | Yes | No |  |  |  |
| 12 | Were limited in the kind of work or other activities | Yes | No |  |  |  |
| 13 | Cut down the amount of time you spent on work or other activities | Yes | No |  |  |  |
| 14 | Had difficulty performing the work or other activities (for example, it took extra effort) | Yes | No |  |  |  |
| GENERAL HEALTH (GH) 1+4 | | | | | | |
| 15 | In general, would you say your health is | Excellent | Very Good | Good | Fair | Poor |
| 16 | I seem to get sick a little easier than other people | Definitely true | Mostly true | Not sure | Mostly false | Definitely false |
| 17 | I am as healthy as anybody I know | Definitely true | Mostly true | Not sure | Mostly false | Definitely false |
| 18 | I expect my health to get worse | Definitely true | Mostly true | Not sure | Mostly false | Definitely false |
| 19 | My health is excellent | Definitely true | Mostly true | Not sure | Mostly false | Definitely false |
| BODILY PAIN（BP）0+2 | | | | | | |
| 20 | How much bodily pain have you had during the past 4 weeks? | None | Very mild | Mild | Moderate Severe | Very severe |
| 21 | During the past 4 weeks, how much did pain interfere with your normal work (including both work outside the home and housework?) | None | Very mild | Mild | Moderate Severe | Very severe |

Thank you very much for your participation and cooperation!

**Appendix B Existing CCRC Projects in Shanghai**

| **Code** | **CCRC projects in Shanghai** | **Year** | **Corporations** |
| --- | --- | --- | --- |
| 1 | Vcan Senior Jade Tower (鸿泰·乐璟会) | 2018 | Yongtai Hongkan Elderly Care Industry Development Group Co., Ltd. |
| 2 | Rui-ci Retirement Community (上实东滩长者社区/上实瑞慈花园) | 2018 | Shanghai Industrial Investment (Holdings) Co., Ltd. |
| 3 | CHERISH-YEARN (亲和源·康桥爱养之家) | 2008 | Cherish-Yearn Co., Ltd. |
| 4 | Wonder Lane (上海申养忘年荟) | 2017 | Shanghai Real Estate Group and Vanke Group |
| 5 | China Taiping International Senior Living Community (梧桐人家国际健康颐养社区) | 2020 | China Taiping Insurance Group |
| 6 | Landlease Ardor Gardens (联实逸浦荟) | 2020 | Lendlease Group |
| 7 | NEY International Evergreen Homeland(新东苑-快乐家园) | 2017 | New East Garden International Investment Group Co., Ltd. |
| 8 | Senior Living L’AMORE-Shanghai Kangqiao (椿萱茂-上海康桥) | 2017 | Sino-Ocean Group |
| 9 | Star Castle (星堡) | 2013 | Shanghai Fosun Group and Fortress Investment Group |
| 10 | Greenland Community – Yishangju (绿地国际康养城-颐尚居) | 2020 | Greenland Holdings Corp. Ltd. |
| 11 | Greenland International Residence Hotel (绿地国际旅居酒店) | 2020 | Greenland Holdings Corp. Ltd. |
| 12 | Yunqi Lanting (云栖兰亭) | 2017 | Dongju Enterprise (Shanghai) Development Co., Ltd. |
| 13 | Xiangshuwan (香树湾) | 2015 | Shanghai Daofu Elderly Care Investment Management Co., Ltd. |
| 14 | Xiangheyuan (祥和源) | 2016 | Shanghai Fuyi Elderly Care Co., Ltd. |
| 15 | Puyue - Limitless Love (璞悦-安居里) | 2017 | Ci Yi Hui Yang (Shanghai) Industrial Co., Ltd. |
| 16 | China Taiping - Wutong Home (中国太平-梧桐人家) | 2019 | China Taiping Insurance Group |
| 17 | Aveo (China) Campus (爱维中国-天地健康城) | 2015 | FKP Property Group (now Aveo Group) and China Tiandi Holdings |
| 18 | Taikang Community-Shenyuan (泰康之家-申园) | 2016 | Taikang Life Insurance Co., Ltd. and Taikang Health Industry Investment Holdings Co., Ltd. |

**Appendix C** **Focused Overview of the Diversity, Features, and Management of 13 Selected CCRCs**

| **Category** | **CCRCs** | **Investment/Development/Operation Model (Entity)** | **Profit Model** | **Building Area/Project Capacity/Current Number of Residents** | **Advantages and Features** |
| --- | --- | --- | --- | --- | --- |
| All-age institution | Aveo (China) Campus | Joint venture between FKP Property Group (now Aveo Group) and China Tiandi Holdings, forming AVEOCHINA subsidiary. Joint development leveraging AVEO’s senior living operation experience and China’s medical network. Operated by Aveo China. | Sales + Leasing Model: 70% sale for quick ROI, 30% leasing with insurance company partnerships for stable long-term returns. | 150,000 sqm; 1,200 units (788 independent living units); approx. 850 residents | -Includes hotel and kindergarten for visitors and residents’ families, creating an all-age community.  -Combines Australian AVEO’s 30+ years of high-end living experience for older adults with local expertise. |
|  | China Taiping - Wutong Home | Funded and developed by China Taiping Insurance Group through China Taiping Investment, and operated by Taiping Pension Industry Investment Management Co. | Sales + Membership Card Model: Combines insurance product sales, membership sales, and elderly care services for diversified profits. Some units for sale to alleviate initial funding pressures; some units available through annuity insurance for permanent, lifetime, or trial stays, with varying usage rights and transfer conditions; offers self-funded and insurance-covered rehabilitation, outpatient, and physical examination services to increase revenue. | 189,000 sqm; 3,500 units; approx. 800 residents | -Invested and built by China Taiping Insurance Group, a Fortune Global 500 company.  -Implements a “migratory bird” living and elderly care model, with residents living in different community locations across 16 cities based on the season (e.g., winter in Hainan, summer in Dalian).  -Annuity insurance provides long-term security for the elderly, covering all community expenses and ensuring the long-term reliability of the life insurance company, offering a unique advantage over other industries.  -Annuity insurance purchase grants “residency rights” (for both parents and children), promoting the establishment of all-age communities. |
|  | Greenland Community - Yishangju | Funded by Greenland Holdings Corporation Limited, developed by Shanghai Greenland Qingcheng Real Estate Co., Ltd., and operated by Shanghai Greenland Health and Wellness Elderly Care Service Co., Ltd. | Membership Card Model: Membership cards to alleviate initial funding pressures, divided into A and B cards. A cards have a fixed price, can be used permanently, inherited, and transferred but not refunded; B cards increase in price with larger unit sizes. Residents pay monthly for utilities and dining services. | 131,000 sqm; 1,000 units; approx. 800 residents | -Invested and built by Greenland Group, a Fortune Global 500 company.  -Provides a “migratory bird” tourism and elderly care model, covering health residences and hotels nationwide.  -Phase III plans to build long-term rental apartments and introduce educational facilities like kindergartens to create an all-age community. |
|  | Yunqi Lanting | Funded by Dongju Enterprise (Shanghai) Development Co., Ltd., developed independently by Dongju Enterprise (Shanghai) Development Co., Ltd., and operated by Shanghai Dongju Elderly Care Service Co., Ltd. | Membership Card Model: Membership cards (permanent, 15-year, and 5-year) grant residency rights; can be inherited, transferred, and repurchased. Short-term rentals include daily, monthly, semi-annual, and annual options. Only property fees are mandatory after moving in. | 16,000 sqm; over 600 units (300 currently open); approx. 300 residents | -Operates on a “health and wellness + travel” model.  -Membership can be inherited and repurchased, offering both investment and residency benefits; flexible short-term rental services available.  -Provides family rooms with children’s play tents and toys, promoting an all-age community. |
| Age homogeneity institution | Taikang Community-Shenyuan | Funded by Taikang Life Insurance Co., Ltd. and Taikang Health Industry Investment Holdings Co., Ltd., developed by Guangnian (Shanghai) Investment Co., Ltd., and operated by Taikang Home Shenyuan (Shanghai) Elderly Care Service Co., Ltd. and Shanghai Taikang Shenyuan Rehabilitation Hospital Co., Ltd. | Membership Card Model: Residency rights obtained through purchasing insurance products (e.g., “Happiness Lifelong Pension Plan”) or direct payment with deposit and monthly fees (housing, living, and other service fees). | 224,000 sqm; 2,100 senior living units (including 200 medical beds); approx.1995 residents | -Invested and built by Taikang Group, a Fortune Global 500 company.  -Pioneer of insurance-based senior care communities in China, approved by the China Insurance Regulatory Commission in 2009.  -Largest senior care community in Shanghai and East China.  -Integrates “pension insurance and senior care community, health insurance and medical system, pension funds and asset management” into three closed loops, achieving an organic combination of “insurance + medical care + asset management”. |
|  | Landlease Ardor Gardens | Fully integrated development, construction, and operation by Lendlease Group. | Membership Card Model: Offers various membership cards and housing options, including long-term (30 years), mid-term (15 years), and short-term (1 year) memberships. Membership cards can be refunded, renewed, paused, or deferred. Monthly service fees apply after move-in. | 9,400 sqm; over 150 units; approx. 30 residents | -Invested and built by Lendlease Group, a Fortune Global 500 company.  -Entirely foreign-invested senior care project; first integrated development model by Lendlease in China.  -Operates as a hotel-style CCRC without the need to purchase property or insurance, using a hotel-style and membership-based senior care model. |
|  | Greenland International Residence Hotel (Greenland Kang Young Hotel) | Funded by Greenland Holdings Corporation Limited | Leveraging Greenland Community - Yishangju, the membership card provides access to care services at all Greenland International Residence Hotels nationwide. | 1000 units; approx.300 residents |  |
|  | NEY International Evergreen Homeland | Funded by New East Garden International Investment Group Co., Ltd. Joint venture with Eaton Service Co. Partnerships with Samsung Seoul Hospital, Samsung Noble County, Paris International Anti-Aging Center, Shuguang Hospital, Baosteel Development, Aikang Group, and collaboration with China Taiping Life Insurance for diverse health and elderly care services. | Membership Card Model + Leasing: Membership cards available for 10, 20, and 30 years with corresponding fees, plus monthly service and utility fees; rental option requires three months’ rent as a deposit, with rent and service/utility fees based on room type. | 150,000 sqm; over 650 units; approx. 80 residents | -Established on Shanghai’s first paid-transfer elderly care land.  -First CCRC in Shanghai designed using the “Shanghai Green Elderly Care Building Evaluation Technical Guidelines”, recognized as “Shanghai’s First Green Elderly Care Community”. |
|  | Puyue - Limitless Love | Ci Yi Hui Yang (Shanghai) Industrial Co., Ltd. | Sale + Leasing: Monthly rent, membership, lump-sum payment. | 69,000 sqm; 300 units (including single and double occupancy); approx. 500 residents | -Open CCRCs community integrated with urban areas, promoting social interaction and integration with the wider population.  -Combines senior CCRC with a “Zen” culture hotel for younger people, meeting the wellness needs of the elderly while attracting younger generations, creating a multi-generational living environment. |
|  | Star Castle | Funded by Star Castle’s own capital. Joint venture between Shanghai Fosun Group and Fortress Investment Group, each holding 50%. Jointly developed and owned by Fosun Group, and operated by a professional team from Fortress Investment Group. | Leasing + Membership Card Model: Quickly recoup funds and ensure stable cash flow. Collaborates with Fosun Pramerica Life Insurance to offer a project linking pension insurance with the senior community. Clients can choose to purchase insurance or a 50-year membership card (usage rights only, inheritable and transferable) for residency. Monthly membership fees and dining costs vary by unit size. The rental system charges monthly fees covering accommodation, dining, housekeeping, 24-hour emergency medical services, and clinic visits, with costs varying by unit type. | 90,000 sqm; Phase I: 220 units, Phase II: 900 units; approx. 600 residents | -First senior care facility in Shanghai with foreign investment license, business license, and elderly care license from the Civil Affairs Bureau.  -Pioneered an all-inclusive rental monthly fee model for CCRCs.  -Combines Chinese and foreign expertise, leveraging Fortress’s 30+ years of high-end senior care experience to introduce the American CCRCs service model to China. |
|  | Xiangshuwan | Fully funded by Shanghai Daofu Elderly Care Investment Management Co., Ltd., a wholly-owned subsidiary of Shanghai Wanfeng Group. | Membership Card Model: Residency rights obtained through membership fees; membership is transferable and inheritable. Monthly service fees vary by unit type after move-in. | 110,000 sqm; approx. 950 units (532 units mainly for active seniors); approx. 300 residents |  |
|  | Xiangheyuan | Fully funded and operated by Shanghai Fuyi Elderly Care Co., Ltd. | Leasing: Includes accommodation and care fees. Accommodation fees vary for double rooms and suites, depending on room type and rental period. Care fees are categorized into daily care, rehabilitation care, and special care, with specific costs based on the type of care. | 36,000 sqm; approx. 485 units (655 beds); approx. 500 residents |  |
| “Embedded” older care facilities within its neighbourhood | Senior Living L’AMORE-Shanghai Kangqiao | Funded by Sino-Ocean Group, managed by Sino-Ocean Elderly Care Management Co., Ltd., and operated in collaboration with Emeritus, Meridian Senior Living (MSL), and Validation Training Institute (VTI). | Leasing: Monthly fees vary based on seniors’ self-care ability and care needs, covering bed fees, care fees, and miscellaneous expenses. | 17,000 sqm; 173 units (343 beds); approx. 200 residents | -Develops embedded CCRCs using a light asset model by leasing and converting suitable buildings into senior apartments with elderly care services.  -Awarded in 2023 as one of the “Top 10 Senior Living Brands in China,” “Best Elderly Care Service Brand,” and “Best Boutique Senior Living Facility.” |

Note: 1. “Number of Residents” refers to the data collected during the survey period (November 2021 to May 2022), provided by community staff and recorded by researchers. 2. “Building Area” refers to the total constructed area of the community, encompassing active health zones, nursing and rehabilitation zones, and shared facilities. Our research primarily focuses on independently living elderly individuals (as specified in the ethics application), who occupy only a subset of the total area. Given the challenges in disaggregating specific building areas and the inclusion of shared facility zones, we present the total building area. 3. The “Membership Card Model” is a unique system currently adopted by major CCRCs in China. It allows residents to obtain living and service rights within the community through the purchase of high-value membership cards, which are typically inheritable and transferable. Depending on the community’s regulations, the cards may offer varying levels of care and services. This system facilitates rapid capital recovery during the initial stages and ensures long-term stable cash flow, making it a primary profit model for many Chinese CCRCs. 4. Specific amounts related to “membership cards” and associated fees, which are subject to frequent changes, are not included in the table; instead, the emphasis is placed on outlining the overall profit model.
